# Ferroptosis in Osteocytes as a Target for Protection Against Postmenopausal Osteoporosis

**DOI:** 10.1002/advs.202307388

**Published:** 2024-01-17

**Authors:** Zengxin Jiang, Guobin Qi, Xuecheng He, Yifan Yu, Yuting Cao, Changqing Zhang, Weiguo Zou, Hengfeng Yuan

**Affiliations:** ^1^ Department of Orthopaedics Shanghai Jiaotong University Affiliated Sixth People's Hospital No. 600 Yishan Road Shanghai 200233 China; ^2^ Institute of Microsurgery on Extremities Shanghai Jiao Tong University Affiliated Sixth People's Hospital Shanghai 200233 China; ^3^ State Key Laboratory of Cell Biology CAS Center for Excellence in Molecular Cell Sciences Shanghai Institute of Biochemistry and Cell Biology Chinese Academy of Sciences University of Chinese Academy of Sciences Shanghai 200031 China

**Keywords:** osteocytes, ferroptosis, postmenopausal osteoporosis, DNA methylation, nuclear factor kappa‐B ligand

## Abstract

Ferroptosis is a necrotic form of iron‐dependent regulatory cell death. Estrogen withdrawal can interfere with iron metabolism, which is responsible for the pathogenesis of postmenopausal osteoporosis (PMOP). Here, it is demonstrated that estrogen withdrawal induces iron accumulation in the skeleton and the ferroptosis of osteocytes, leading to reduced bone mineral density. Furthermore, the facilitatory effect of ferroptosis of osteocytes is verified in the occurrence and development of postmenopausal osteoporosis is associated with over activated osteoclastogenesis using a direct osteocyte/osteoclast coculture system and glutathione peroxidase 4 (GPX4) knockout ovariectomized mice. In addition, the nuclear factor erythroid derived 2‐related factor‐2 (Nrf2) signaling pathway is confirmed to be a crucial factor in the ferroptosis of osteocytic cells. Nrf2 regulates the expression of nuclear factor kappa‐B ligand (RANKL) by regulating the DNA methylation level of the RANKL promoter mediated by DNA methyltransferase 3a (Dnmt3a), which is as an important mechanism in osteocytic ferroptosis‐mediated osteoclastogenesis. Taken together, this data suggests that osteocytic ferroptosis is involved in PMOP and can be targeted to tune bone homeostasis.

## Graphical abstract

Ferroptosis of osteocytes is a major factor implicated in the pathogenesis of osteoporosis. Ferroptotic osteocytes play a critical role in modulating bone loss through the regulation of osteoclast‐mediated bone resorption. The transcriptional regulation of Dnmt3a by Nrf2 leads to the methylation of the RANKL promoter, serving as a crucial molecular mechanism by which ferroptotic osteocytes promote osteoclast formation and differentiation.

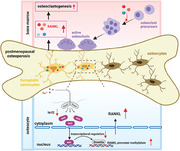

## 1 Introduction

Osteoporosis is a common bone disease characterized by low bone mass and bone microstructure failure. The number of adults in the United States with osteoporosis and low bone mass is projected to increase to ≈71 million by 2030.^[^ ^1^ ^]^ Among the risk factors that contributes to the development of osteoporosis in elderly women is estrogen deficiency. Osteoporotic fractures are a serious health problem, particularly for aging, postmenopausal women. Prevention and treatment of postmenopausal osteoporosis (PMOP) is essential, especially in elderly women who have an incidence of osteoporotic fractures approaching 50%.^[^ ^2^ ^]^

After menopause, estrogen levels suddenly decrease, and the roles of estrogen in promoting apoptosis of mature osteoclasts, activating osteoprotegerin and inhibiting receptor activator of the nuclear factor kappa‐B ligand (RANKL) and RANKL activities are blunted, leading to hyperactive osteoclastogenesis and a subsequent imbalance of bone remodeling, finally resulting in bone loss in postmenopausal women.^[^ ^3^, ^4^, ^5^, ^6^ ^]^ Although current therapies targeting osteoclasts are beneficial, they are still far from sufficient.^[^ ^7^, ^8^ ^]^ Therefore, a better understanding of the underlying mechanisms regulating bone loss is needed. The signals of targeted recruitment and activation of osteoclast precursors mainly come from osteocytes at the beginning of the bone remodeling cycle.^[^ ^9^, ^10^ ^]^ Osteocytes, the most numerous cells in the bone, play an essential role in regulating bone homeostasis. Osteocytes form complex networks through ramifying dendritic processes, which enables osteocytes to detect and respond to mechanical strain and signaling molecules, including growth factors, cytokines, and hormones. Through this network, osteocytes can regulate the formation and function of osteoblasts and osteoclasts to maintain bone homeostasis and metabolism.^[^ ^11^, ^12^, ^13^ ^]^ In addition, osteocytes secrete a variety of cytokines and hormones, including nitric oxide, prostaglandin E2, adenosine triphosphate, sclerostin, Dickkopf‐related protein 1, and RANKL.^[^ ^14^, ^15^ ^]^ Given the large number of osteocytes and rich blood vessels in bone tissues, osteocytes, as endocrine cells, should be studied in biological activities. Growing evidence indicates that abnormal osteocyte functions and excessive cell death underlie the pathophysiology of several skeletal disorders.^[^ ^16^, ^17^, ^18^ ^]^ However, the effects of systemic metabolic alterations caused by a decrease in estrogen on osteocytes are insufficiently understood. The mechanisms by which these alterations regulate osteoclasts in a coordinated manner through osteocytes require further investigation.

Ferroptosis is an iron‐dependent, nonapoptotic regulated cell death, and reactive oxygen species (ROS) generated during the excess iron‐mediated Fenton reaction cause peroxidation of phospholipids at the plasma membrane and ultimately lead to ferroptosis. Glutathione peroxidase 4 (GPX4), a selenoprotein glutathione peroxidase with pleotropic functions, is an essential negative regulator of ferroptosis.^[^ ^19^, ^20^, ^21^ ^]^ Serum ferritin levels increase significantly in postmenopausal women, which has been confirmed to be closely related to the decrease in bone mineral density.^[^ ^22^, ^23^ ^]^ Elevated iron levels likely indicate ferroptosis in osteocytes. Some studies have already noted the close relationship between ferroptosis of osteocytes and bone homeostasis in diabetic osteoporosis.^[^ ^24^ ^]^ But the pathophysiology in diabetic osteoporosis is different from that in PMOP. More importantly, prior investigations have primarily concentrated on the mechanisms that leads cells to undergo ferroptosis, with scarce deliberation on the mechanisms through which ferroptosis of osteocytes instigates bone loss. Currently, within the field of postmenopausal osteoporosis research, only a few studies indicate an increased ferroptosis of circulating monocytes using a bioinformatics analysis.^[^ ^25^ ^]^ It is still unclear whether ferroptosis of osteocytes is involved in PMOP and the mechanisms responsible for osteocytic ferrotosis‐induced bone loss remain poorly understood.

Here, we show that ferroptosis of osteocytes occurs in mice with ovariectomy (OVX)‐induced osteoporosis, which is mainly due to iron accumulation in bone tissues. We verified the facilitatory effect of ferroptosis of osteocytes in the occurrence and development of postmenopausal osteoporosis was associated with over activated osteoclastogenesis. We found that the Nrf2 signaling pathway is the core regulator of ferroptosis in osteocytes. Inhibition of Nrf2 resulted in ferroptosis of osteocytes in a high iron environment. In addition, Nrf2 suppression directly led to high expression of RANKL in osteocytes, which is a critical reason for the osteocytes‐mediated excessive activation of osteoclasts. We elucidated the mechanism that Nrf2 suppression upregulated RANKL in the process of osteocytic ferroptosis. Nrf2 inhibition downregulated DNA methyltransferase 3a (Dnmt3a) at the transcriptional level, leading to a decrease in DNA methylation levels of the RANKL promoter and ultimately upregulating RANKL in osteocytes to promote osteoclast activation.

## 2 Results

### 2.1 Iron Overload Induces Ferroptosis of Osteocytes in OVX Mice

**Figure 1:**
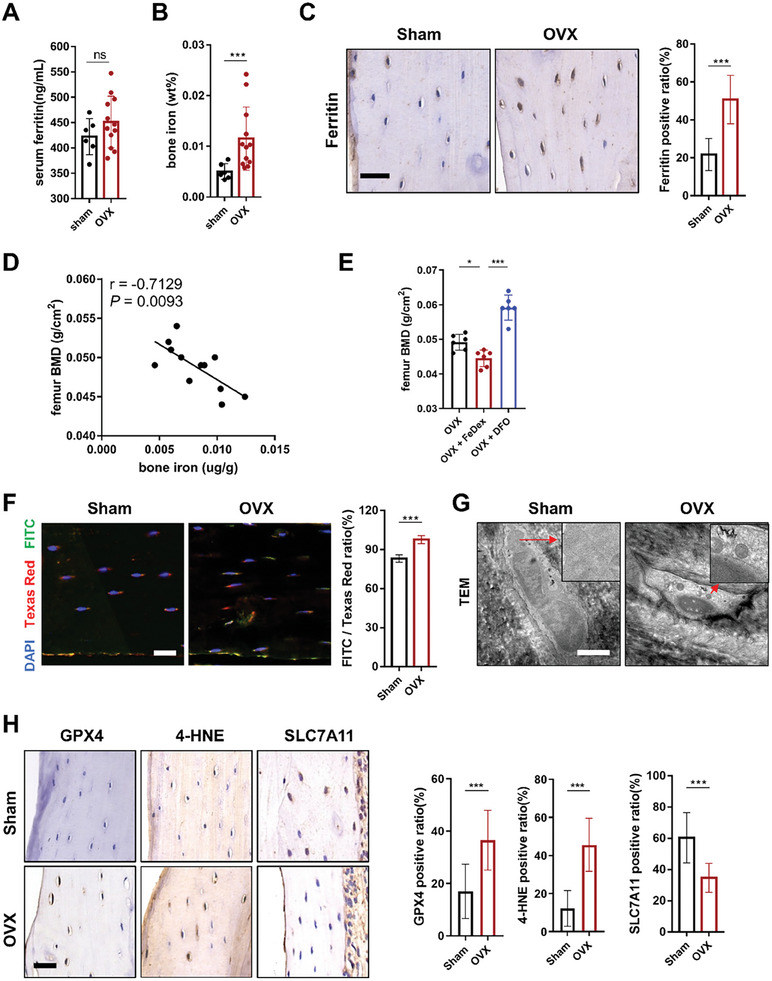
The increase in bone iron levels correlates with bone loss in ovariectomized mice. A) Serum ferritin levels determined by ELISA kits from the sham and ovariectomized mice (sham, *n* = 6; OVX, *n* = 12). B) Bone iron content determined by ICP from the sham and ovariectomized mice (sham, *n* = 6; OVX, *n* = 12). C) Representative images of IHC staining for detecting ferritin and quantification (scale bar, 20 µm). Five randomly selected viewing fields were evaluated per section, and 6 mice were evaluated per group. D) Correlation analysis revealed that the iron level in bone tissues (femur diaphysis) was negatively associated with BMD. E) Dual‐energy X‐ray absorptiometry (DXA) was performed to analyze the bone mineral density (BMD) of femurs in mice. The analyzed area is shown in the schematic diagram. Fedex treatment decreased the BMD of femurs in OVX mice while DFO treatment alleviated the decrease in BMD of femurs in OVX mice (*n* = 6/group). F) Measurement of the level of lipid peroxidation in osteocytes of sham and OVX mice using BODIPY 581/591 fluorescence staining (*n* = 6/group, red: normal lipids; green: oxidized lipids; blue: cell nuclei). Scale bar: 20 µm. G) Transmission electron microscopy was used to observe the morphological changes in mitochondria of osteocytes in the femur of sham and OVX mice. Scale bar: 2 µm. H) Representative images of IHC staining for detecting GPX4, 4‐HNE, SLC7A11 and quantification (scale bar, 20 µm). Five randomly selected viewing fields were evaluated per section, and 6 mice were evaluated per group. Data are represented as the mean ± SD. *P < 0.05, ***P < 0.001, ns = not significant. Two‐tailed paired t test, ANOVA with post‐hoc Tukey–Kramer test or Pearson correlation.

Postmenopausal women are prone to iron accumulation after estrogen withdrawal, with an increased incidence of osteoporosis. We first induced a mouse model of osteoporosis by ovariectomy (OVX) and investigated iron levels. Serum ferritin, as the main existing form of iron in the body, was not significantly different from that in the sham group (**Figure** **1**A). Then, we examined the iron content in the bone tissue by inductively coupled plasma (ICP) analysis. Interestingly, the results showed that the iron content of trabecular and cortical bone was significantly increased in the ovariectomized mice (Figure 1B). Consistent with the ICP data, immunohistochemical staining analysis showed that the ovariectomized mice had a significant increase in ferritin expression in the bone tissue (Figure 1C). Correlation analysis further revealed that the iron level was negatively associated with femur bone mineral density (BMD) (Figure 1D). After that, we treated the mice with intraperitoneal injection of iron dextran (FeDex) and found that iron could further decrease BMD, while deferoxamine (DFO), an extracellular iron chelator, could alleviate bone loss in the ovariectomized mice (Figure 1E).

Next, ferroptosis was confirmed to be the predominant form of death in osteocytic cells in a high‐iron environment. We adopted the Ocy454 cell line for cellular experiments because these cells can differentiate into late‐stage osteocytes at 37 °C and closely recapitulate the phenotype of primary cells.^[^ ^26^ ^]^ In detail, cell viability was first examined by CCK‐8 assays, and the results showed that only Lip‐1 or Fer‐1, which are well‐established and specific ferroptosis inhibitors, could significantly decrease cell death, while Z‐VAD‐FMK (an inhibitor of apoptosis) had no obvious effects. In addition, necrostatin‐1 (Nec‐1, an inhibitor of necroptosis) showed a certain effect although not statistically different, possibly due to the inhibition of TNF‐α, which interfered with ferroptosis (Figure S1A, Supporting Information). In addition, the cell morphology was observed by TEM, and ferric ammonium citrate (FAC)‐treated Ocy454 cells showed obvious shrinkage of mitochondria (Figure S1B, Supporting Information). Moreover, DCFH‐DA staining was used to determine the level of ROS, and the results showed that FAC could increase the ROS level (Figure S1C, Supporting Information). Next, we aimed to test the lipid peroxidation of Ocy454 cells after FAC treatment with C11‐BODIPY staining, and the results showed that the mean ratio of green/red fluorescence was increased (Figure S1D,E, Supporting Information). Additionally, Western blot results showed that after FAC treatment, the levels of GPX4, 4‐HNE, Ferritin Light Chain and Ferritin Heavy Chain were significantly increased, while the level of SLC7A11 was significantly reduced in the Ocy454 cells (Figure S1F, Supporting Information). FerroOrange staining was used to determine the cellular Fe^2+^ levels. The results showed that Fe^2+^ levels increased in the Ocy454 cells exposed to FAC (Figure S1G, Supporting Information). In contrast, these alterations could be reversed by liproxstatin‐1 (Lip‐1) or ferrostatin‐1 (Fer‐1) treatment.

Consistent with the in vitro results, ferroptosis of osteocytes was also observed in OVX mice. In detail, with C11‐Bodipy staining, we found that osteocytes exhibited obvious lipid peroxidation in the ovariectomized mice (Figure 1F). The results of FerroOrange staining also showed increased Fe^2+^ levels in the osteocytes of OVX mice (Figure S2, Supporting Information). Moreover, the mitochondria in the cells were significantly shrunken, as observed by TEM, which manifested as decreased volume, increased density and disappearance of mitochondrial cristae, typical changes of ferroptosis (Figure 1G). In addition, 4‐HNE and SLC7A11, as the classic indicators of lipid peroxidation metabolites, was reported to be increased and decreased in ferroptotic cells, separately. As expected, we found increased expression of 4‐HNE and decreased expression of SLC7A11 in osteocytes with immunohistochemical staining, which provided evidence of ferroptosis in osteocytes from a lipid perspective. These data suggest that osteocytes undergo ferroptosis in the ovariectomized mice. We further detected the level of GPX4. In contrast, GPX4 was highly expressed in the osteocytes of the ovariectomized mice (Figure 1H).

**Figure 2:**
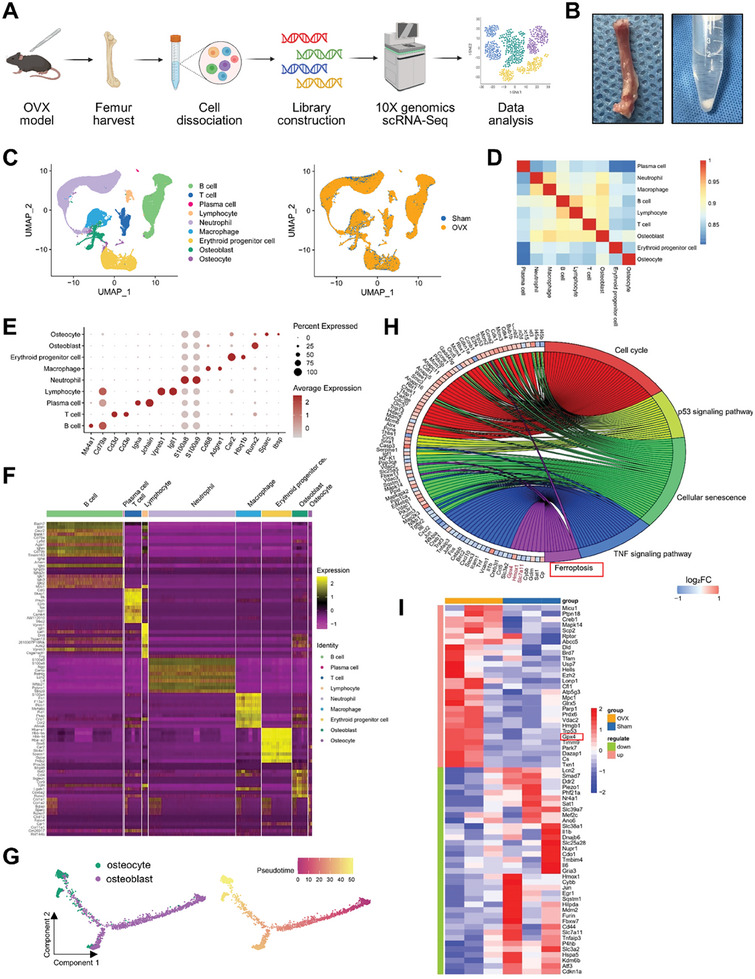
Single‐cell sequencing of mouse femurs showed that osteocytes in OVX mice undergo ferroptosis. A) Schematic workflow of the experimental strategy. B) Photos of the process of harvesting mouse femur tissue and cell separation. C) UMAP dimensional reduction visualizations all samples. D) Heatmap showing the pairwise correlations. E) Dot plot showing the expression of specific signatures in identified cell types. F) Heatmap showing the top 10 marker genes for the various clusters. G) Monocle pseudotime trajectory analysis of the osteocyte and osteoblast subsets. H) KEGG enrichment analysis of differentially expressed genes in osteocytes in sham and OVX mice. I) Heatmap of differentially expressed ferroptosis‐related genes. Ferroptosis‐related genes were obtained from the FerrDb ferroptosis database.

Subsequently, we used single‐cell sequencing technology to analyze distinct functional states and transcriptome expression of sham and OVX mouse femurs (**Figure** **2**A,B). We annotated all cells based on the expression of enrichment genes in each cluster (Figure 2C,D). Representative markers for osteocytes, osteoblasts, macrophages,T cells, B cells, lymphocytes, plasma cells, neutrophils and erythroid progenitor cells were revealed. Specifically, the following clusters were identified: 1) osteocytes (Sparc/Ibsp), 2) osteoblasts (Runx2), 3) macrophages (Cd68/Adgre1), 4) T cells (Cd3d/Cd3e), 5) B cells (Ms4a1/Cd79a), 6) lymphocytes (Vpreb1/Igll1), 7) plasma cells (Igha/Jchain), 8) neutrophils (S100a8/S100a9) and 9) erythroid progenitor cells (Car2/Hbq1b) (Figure 2E; S3A, Supporting Information). Osteocytes, osteoblasts, and macrophages are the focus of this study. The first 10 upregulated genes from these 9 clusters were used to create a heatmap (Figure 2F). Osteocytes are predominantly derived from osteoblasts. To explore the potential transformation between different cell types and visually depict the differentiation paths, the Monocle method was used to determine the pseudotemporal order between the clusters of cells annotated as oteocytes and osteoblasts (Figure 2G). The results indicated the differentiation of osteoblasts into osteocytes, further confirming the reliability of cell annotation. We performed Kyoto Encyclopedia of Genes and Genomes (KEGG) enrichment analysis on the differentially expressed genes (DEGs) in osteocytes from sham and OVX mice (Figure 2H; S3B, Supporting Information). The results of KEGG enrichment analysis indicate a significant enrichment of ferroptosis, suggesting osteocytes of OVX mice underwent ferroptosis. A total of 483 ferroptosis‐related genes were obtained from the FerrDb ferroptosis database. Ferroptosis‐related genes with differential expression based on the results of single‐cell sequencing were screened. Based on the results of single‐cell sequencing, ferroptosis‐related genes with differential expression were screened and shown in the form of a heatmap (Figure 2I). Among these, the upregulation of GPX4, which is a central regulator of ferroptosis, in osteocytes of OVX mice was of interest.

### 2.2 Osteocytic Ferroptosis Contributed to Bone Loss in the Ovariectomized Mice

To further elucidate the function of osteocytic ferroptosis in bone homeostasis, we generated a GPX4 conditional knockout mouse model by crossing GPX4*^fl/fl^* mice with Dmp1*^Cre+^* mice. The Dmp1*^Cre+^*; GPX4*^fl/fl^* mouse construction strategy is shown in Figure S4A (Supporting Information). Genotyping of the GPX4*^fl/fl^* and WT mice is shown in Figure S4B (Supporting Information). The PCR results confirmed the deletion of GPX4 in osteocytes (Figure S4C, Supporting Information). After OVX modeling, the Dmp1*^Cre+^*; GPX4*^fl/fl^* mice had more severe lipid peroxidation (Figure S4D, Supporting Information), suggesting that ferroptosis was aggravated under menopausal conditions.

**Figure 3:**
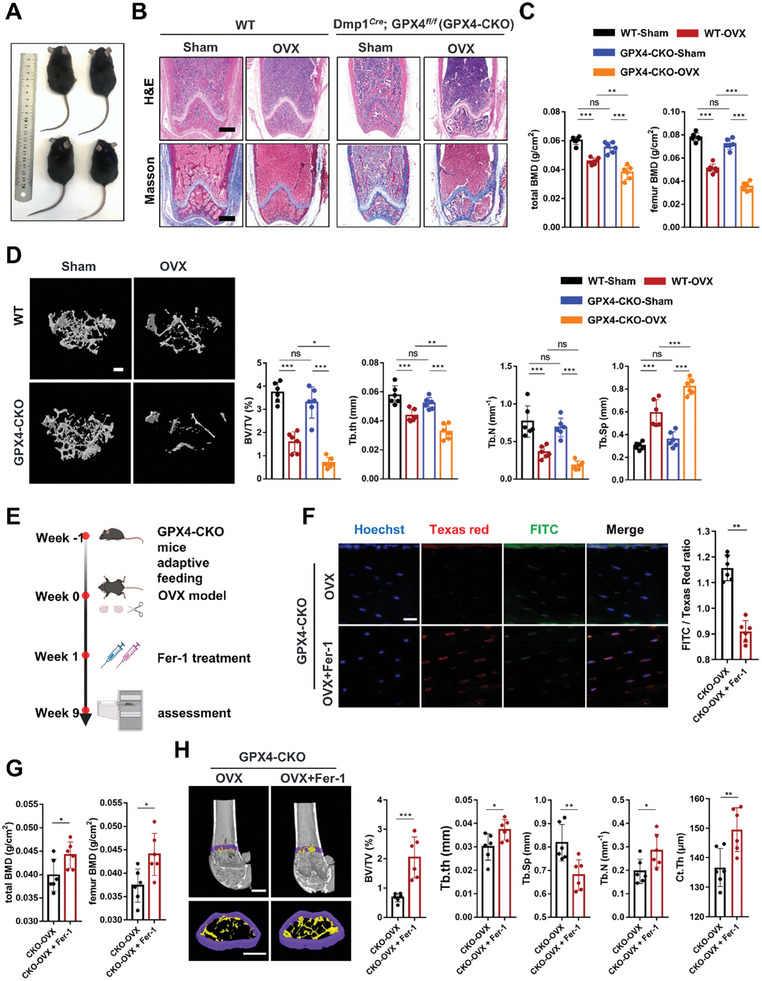
Ferroptosis of osteocytes causes bone loss in OVX mice. A) Representative photos of each group of mice. B) Representative HE and Masson staining of distal femurs in different mice (scale bar, 500 µm). C) Femur BMD and total BMD of each group of mice were assessed using DXA (*n* = 6). D) Representative µ‐CT images of trabecular bone in the distal femurs. (scale bar, 200 µm). Distal femur BV/TV, Tb.Th, Tb. N, and Tb.Sp were measured by µ‐CT in mice in each group (*n* = 6). E) Schematic showing the experimental protocol for 8 weeks of Fer‐1 injections in GPX4‐CKO mice. F) Lipid peroxidation was determined by C11‐BODIPY 581/591 staining from GPX4‐CKO mice treated with and without Fer‐1. The fluorescence intensity of the green/red ratio was quantified (*n* = 6, scale bar, 20 µm, red: normal lipids; green: oxidized lipids; blue: cell nuclei). G) Femur BMD and total BMD of the OVX‐Dmp1^Cre^; GPX4*^fl/fl^* mice treated with and without Fer‐1 (*n* = 6/group). H) Representative µ‐CT images of trabecular bone in the distal femurs (scale bar, 200 µm). Distal femur BV/TV, Tb.Th, Tb. N, Tb.Sp, and Ct.Th were analyzed (*n* = 6/group). GPX4‐CKO: Dmp1^Cre^; GPX4*^fl/fl^* mice. Data are represented as the mean ± SD. *P < 0.05, **P < 0.01, ***P < 0.001, ns = not significant. ANOVA with post‐hoc Tukey–Kramer test.

We then examined osteoporosis‐related indicators. A representative photo of the Dmp1*^Cre+^*; GPX4*^fl/fl^* mice is displayed in **Figure** **3**A. H&E and Masson staining showed that the density of trabecular bone in the distal femur of the Dmp1*^Cre+^*; GPX4*^fl/fl^* mice was significantly reduced, and the trabecular bone was diluted and thinner compared with WT littermates after OVX modeling (Figure 2B). The results of dual‐energy X‐ray detection showed that the total BMD and femoral BMD of the Dmp1*^Cre+^*; GPX4*^fl/fl^* mice slightly reduced than those of the WT group despite the difference was not statistically significant. However, after OVX modeling, the total BMD and femoral BMD of the Dmp1*^Cre+^*; GPX4*^fl/fl^* mice severely decreased (Figure 3C). In addition, the µ‐CT results showed that BV/TV, Tb.Th and Tb.N of the Dmp1*^Cre+^*; GPX4*^fl/fl^* mice were significantly decreased, while Tb.Sp was significantly increased after OVX modeling, suggesting that osteocytic ferroptosis is an important factor in the development of postmenopausal osteoporosis (Figure 3D). Ovariectomized Dmp1*^Cre+^*; GPX4*^fl/fl^* mice showed the most serious osteocytic ferroptosis and bone loss.

We next investigated whether anti‐ferroptosis treatment by injection of Fer‐1 had a remedial effect on ovariectomized Dmp1*^Cre+^*; GPX4*^fl/fl^* mice considering the most serious osteocytic ferroptosis and bone loss. we have carried out some detection on mice. Schematic showing the experimental protocol for 8 weeks of Fer‐1 injections (Figure 3E). As expected, Fer‐1 could effectively inhibit lipid peroxidation, prevent mitochondrial alterations and reduce Fe^2+^ levels in osteocytes within bone tissue, indicating that Fer‐1 could exert anti‐ferroptosis effects in vivo (Figure 3F; Figure S5A,B, Supporting Information). The histological analysis of BMD in mice further confirmed that inhibition of osteocyte ferroptosis could alleviate bone loss (Figure 3G). Furthermore, the µ‐CT analysis showed that BV/TV, Tb.Th, Tb.N, and Ct.Th of the Dmp1*^Cre+^*; GPX4*^fl/fl^* mice with OVX modeling were significantly increased, while Tb.Sp was significantly decreased after Fer‐1 treatment (Figure 3H). Inhibiting ferroptosis of osteocytes effectively alleviated bone loss in OVX mice, confirming the important role of osteocytic ferroptosis in postmenopausal osteoporosis.

### 2.3 Osteocytic Ferroptosis Promoted Osteoclastic Bone Resorption

**Figure 4:**
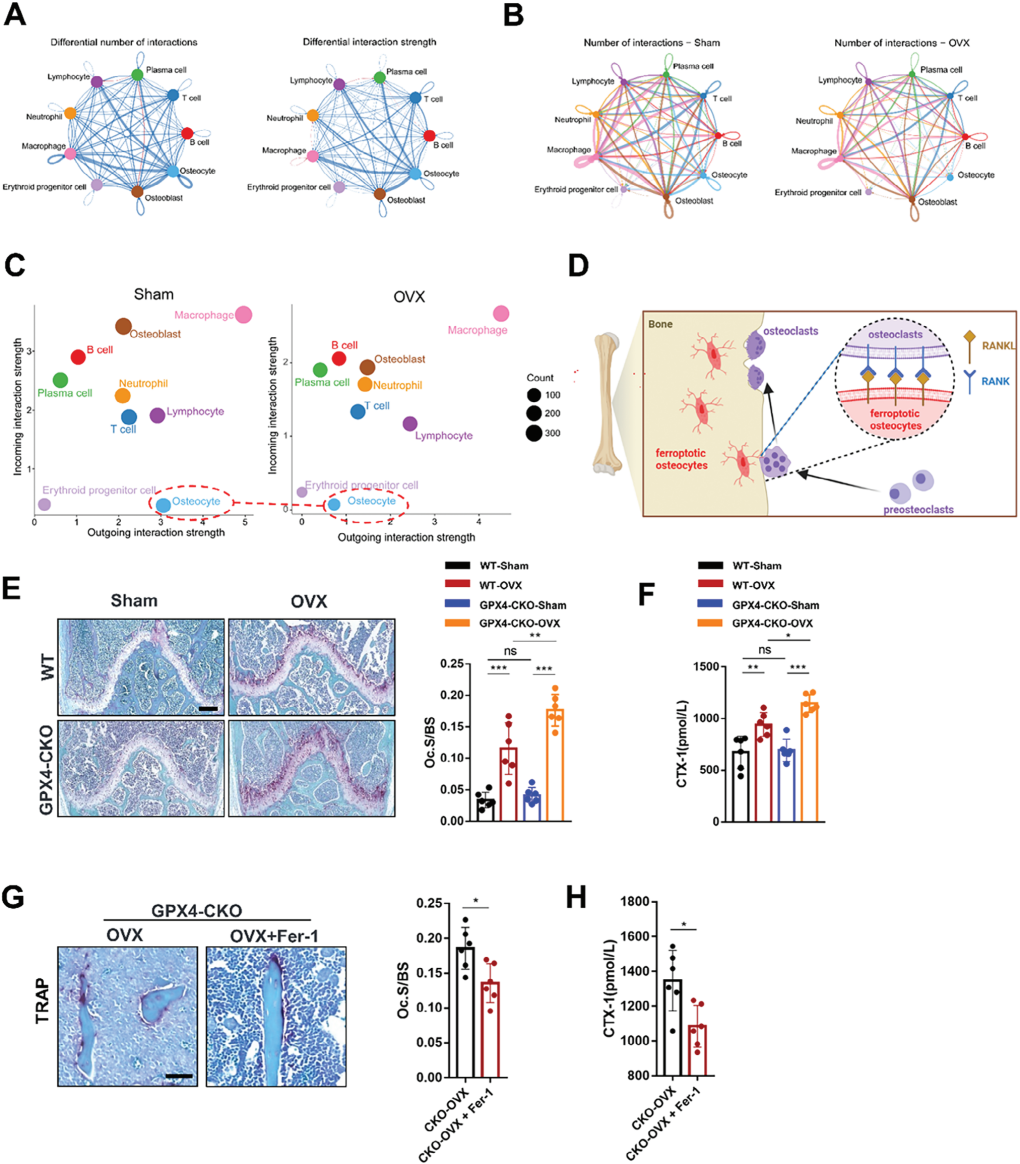
Ferroptosis of osteocytes leads to hyperactive osteoclastogenesis. A) Circle plots showing the differential number of interactions and differential interaction strengths of interactions in femurs between sham and OVX mice. B) Circle plots showing the numbers of interactions in each group. C) Bubble plot showing the incoming and outgoing interaction strength for each cell cluster in sham and OVX mice. D) Schematic showing osteoclastogenesis promotion by ferroptosis of osteocytes. E) Representative images of TRAP staining in the distal femoral metaphysis of mice in each group (scale bar, 200 µm). The osteoclast surface/bone surface (Oc.S/BS) values in the distal femurs of mice in each group (*n* = 6). F) Serum CTX‐1 levels of mice in each group (*n* = 6/group). G) Representative images of TRAP staining of trabecular bone in the distal femurs of the OVX‐Dmp1^Cre^; GPX4*^fl/fl^* mice treated with and without Fer‐1 (scale bar, 50 µm) and quantification of the trabecular osteoclast surface to BS ratio (Oc.S/BS) (*n* = 6/group). H) Serum CTX‐1 levels of the OVX‐Dmp1^Cre^; GPX4*^fl/fl^* mice treated with and without Fer‐1 (*n* = 6/group). GPX4‐CKO: Dmp1^Cre^; GPX4*^fl/fl^* mice. Data are represented as the mean ± SD. *P < 0.05, **P < 0.01, ***P < 0.001, ns = not significant. Two‐tailed Student's t test.

Osteocytes engage in complex signaling processes to facilitate communication with various types of cells, including osteoblasts and osteoclasts, enabling regulation of skeletal system growth, repair, and remodeling. Hence, to investigate the interaction between different cell types in sham and OVX mice, we performed further analysis by CellChat. The results indicated that the strength of interactions and the numbers of intercations between osteocytes and other cells was significantly altered (**Figure** **4**A,B). Especially, there was a significant decrease in the strength of outgoing interaction from osteocytes (Figure 4C). Postmenopausal osteoporosis is a high‐turnover type of osteoporosis characterized by hyperactivated osteoclasts. Therefore, we hypothesized that osteocytic ferroptosis‐mediated bone loss is associated with osteoclast activation (Figure 4D). The robust endocrine function of osteocyts serves as a crucial mechanism for regulating cells on the bone surface and in distant organs. Hence, we first detected the osteoclastogenesis‐related cytokines secreted by osteocytes and found that the levels of RANKL, M‐CSF, IL‐1β, IL‐6, and TNF‐α secreted by Ocy454 cells after FAC treatment were significantly increased, and they could be inhibited by Fer‐1 and Lip‐1 (Figure S6A, Supporting Information). In addition to these soluble cytokines, we further stained Ocy454 with RANKL by immunofluorescence staining, and the results showed that RANKL expression was increased significantly as well (Figure S6B, Supporting Information). Next, we established a direct coculture system to explore the effect of osteocytic ferroptosis on osteoclasts (Figure S6C, Supporting Information). Moreover, FAC‐treated Ocy454 cells could increase the number of Tartrate‐resistant acid phosphatase (TRAP)‐positive cells and the area of bone resorption pits. In contrast, after Fer‐1 or Lip‐1 treatment, the number of TRAP‐positive cells the area of bone resorption pits all decreased to a certain extent (Figure S6D, Supporting Information).

We next explored the relative contributions of osteoblast and osteoclast activity to the bone loss observed in the Dmp1*^Cre+^*; GPX4*^fl/fl^* mice. TRAP‐positive osteoclasts were increased in the distal femur compared with those of the WT mice after OVX modeling (Figure 4E), indicating that osteocytic ferroptosis could activate osteoclasts to promote bone resorption. In addition, serum CTX‐1 was measured as a marker of systemic bone resorption, and the serum CTX‐1 levels in the Dmp1*^Cre+^*; GPX4*^fl/fl^* mice were significantly increased compared with those in the WT mice after OVX modeling (Figure 4F). Interestingly, inhibiting ferroptosis of osteocytes could significantly reduce the TRAP‐positive osteoclasts in ovariectomized Dmp1*^Cre+^*; GPX4*^fl/fl^* mice (Figure 4G), and the serum CTX‐1 levels were significantly decreased as well (Figure 4H).

Subsequently, we explored the impact of osteocyte ferroptosis on osteoblasts. The results of alkaline phosphatase (ALP) and alizarin red S (ARS) staining in the coculture model of Ocy454 and osteoblasts showed that ferroptotic Ocy454 cells had no significant effect on the osteogenic and mineralizing abilities of MC3T3‐E1 cells (Figure S7A, Supporting Information). Consistently, this conclusion was supported by the dual calcein labeling study in vivo. No difference was observed in MS/BS and MAR between the Dmp1*^Cre+^*; GPX4*^fl/fl^* and the WT mice under the same conditions (with or without OVX), suggesting that osteocyte ferroptosis had no appreciable effect on osteoblast‐mediated bone formation (Figure S7B, Supporting Information). These data indicate that osteocytic ferroptosis participate in the development of osteoporosis by activating osteoclast‐mediated bone resorption instead of inhibiting osteoblast‐mediated bone formation.

### 2.4 Nrf2 Signaling is a Key Mechanism for Osteocytic Ferroptosis

**Figure 5:**
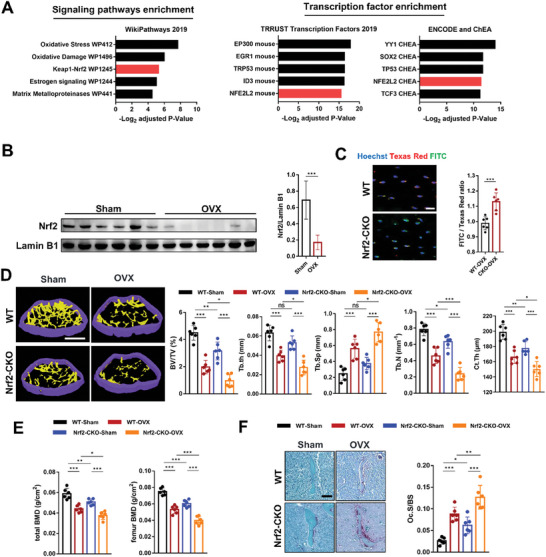
Nrf2 inhibition contributed to bone loss by promoting ferroptosis of osteocytes and subsequent hyperactive osteoclastogenesis. A) Differential expression genes were subjected to Enrichr analysis. Overrepresented signaling pathways and enriched transcription factors are shown. B) Representative Western blot and quantification of the protein level of Nrf2 in bone tissues (femoral shaft) of the sham and OVX mice (*n* = 6/group). C) Lipid peroxidation was determined by C11‐BODIPY 581/591 staining from the WT and Nrf2‐CKO mice (*n* = 6, red: normal lipids; green: oxidized lipids; blue: cell nuclei). scale bar, 20 µm. D) Representative µ‐CT images of distal femurs (scale bar, 250 µm). Distal femur BV/TV, Tb.Th, Tb. N, Tb.Sp and Ct.Th of mice in each group were measured by µ‐CT (*n* = 6/group). E) Total BMD and femur BMD of the sham‐WT, OVX‐WT, sham‐Dmp1^Cre^;Nrf2*^fl/fl^* and OVX‐Dmp1^Cre^;Nrf2*^fl/fl^* mice (*n* = 6/group). F) Representative images of TRAP staining of trabecular bone in the distal femurs of the sham‐WT, OVX‐WT, sham‐Dmp1^Cre^; Nrf2*^fl/fl^* and OVX‐ Dmp1^Cre^; Nrf2*^fl/fl^* mice (scale bar, 50 µm) and quantification of the trabecular osteoclast surface to BS ratio (Oc. S/BS) (*n* = 6/group). Nrf2‐CKO: Dmp1^Cre^; Nrf2*^fl/fl^* mice. Data are represented as the mean ± SD. *P < 0.05, **P < 0.01, ***P < 0.001, ns = not significant. Two‐tailed paired t test or ANOVA with post‐hoc Tukey–Kramer test.

To further explore the transcriptional regulatory networks involved in the ferroptosis process in osteocytes, we conducted the “Enrichr” analysis on the DEGs in osteocytes from sham and OVX mice and identified the Nrf2 antioxidant cellular defense pathway as a top candidate pathway (**Figure** **5**A). These two independent analyses suggest that Nrf2 signaling is a critical pathway in the ferroptosis of osteocytes. Additionally, it is worth noting that GPX4 knockdown led to Nrf2 overexpression in mice, which provided an explanation for the absence of significant bone loss in CKO mice prior to the initiation of OVX modeling (Figure S8A, Supporting Information). Inhibition of Nrf2 by ML385 induced ferroptosis in osteocytes of GPX4‐CKO mice, further confirming the protective role of Nrf2 from ferroptosis in GPX4 knockdown osteocytes (Figure S8B,C, Supporting Information).

To validate the above results, we used Nrf2 small‐interfering RNA (siRNA) and Dimethyl fumarate (DMF, a Nrf2 agonist) to delineate the Nrf2‐mediated regulation of ferroptosis in osteocytes. The Western blot results showed that FAC treatment inhibited the nuclear translocation of Nrf2 and prevented the downstream NQO1 and HO‐1 expression but enhanced Keap1 expression (Figure S9A, Supporting Information), which confirmed the knockdown and activation of Nrf2. Promoting the nuclear translocation of Nrf2 effectively prevented lipid peroxidation while inhibiting Nrf2 promoted lipid peroxidation in Ocy454 cells exposed to FAC (Figure S9B, Supporting Information). In addition, inhibiting the nuclear translocation of Nrf2 increased the 4‐HNE level and reduced the SLC7A11 level in Ocy454 cells exposed to FAC. These changes were reversed by Nrf2 activation (Figure S9C, Supporting Information). Schematic of Nrf2 function in the regulation of ferroptosis in osteocytes was shown in Figure S9D, Supporting Information. Next, we investigated the impact of osteocytes on osteoclasts following the regulation of Nrf2 using a direct coculture system as described above. As expected, TRAP staining results showed that coculture with Nrf2 siRNA‐transferred ferroptotic osteocytes could increase the number of TRAP‐positive cells. Promoting the nuclear translocation of Nrf2 in FAC‐treated Ocy454 cells significantly prevented the formation of osteoclasts (Figure S9E, Supporting Information).

We next explored the potential function of Nrf2 in ferroptotic osteocytes at the animal level. The downregulation of Nrf2 in the osteocytes of OVX mice was also confirmed by Western blotting (Figure 5B). We then generated an Nrf2 conditional‐knockout mouse model by crossing Nrf2*^fl/fl^* mice with Dmp1*^Cre+^* mice. The Dmp1^Cre^; Nrf2*^fl/fl^* mouse construction strategy is shown in Figure S10A (Supporting Information). Genotyping of the Nrf2*^fl/fl^* and WT mice is shown in Figure S10B (Supporting Information). The PCR results confirmed the deletion of Nrf2 in osteocytes (Figure S10C, Supporting Information). Deletion of Nrf2 in osteocytes led to obvious shrinkage of mitochondria (Figure S10D, Supporting Information). Even though, deletion of Nrf2 in osteocytes showed slight high Fe2+ levels, there was no statistical difference. This could be attributed to the absence of OVX modeling (Figure S10E, Supporting Information). The lipid peroxidation was significantly increased in the Dmp1^Cre^; Nrf2*^fl/fl^* mice, indicating an increased level of osteocytic ferroptosis (Figure 5C). The µ‐CT results showed that BV/TV, Tb.N, and Ct.Th of the Dmp1^Cre^; Nrf2*^fl/fl^* mice were significantly decreased in the Dmp1^Cre^; Nrf2*^fl/fl^* mice (Figure 5D). Further analysis of BMD showed that the total BMD and femoral BMD of the Dmp1^Cre^; Nrf2*^fl/fl^* mice were significantly lower than those of the WT group (Figure 5E). Similarly, the deterioration of the trabecular microarchitecture and decrease in BMD were exacerbated after OVX modeling. The results of TRAP staining showed that Oc.S/BS was significantly increased in the Dmp1*^Cre+^*; Nrf2*^fl/fl^* mice compared with the WT mice under the same conditions (with or without OVX) (Figure 5F), suggesting that Nrf2 knockout in osteocytes promoted osteoclast‐mediated bone resorption. We further treated Dmp1*^Cre+^*; Nrf2*^fl/fl^* mice with Fer‐1 and found that anti‐ferroptosis treatment could significantly increase the total BMD and femur BMD (Figure S11A, Supporting Information) and inhibit deterioration of bone microarchitecture inhibits deterioration of bone microarchitecture (Figure S11B,C, Supporting Information), indicating that Nrf2 signaling is a key mechanism for osteocytic ferroptosis. The results above suggesting that Nrf2 knockout in osteocytes promoted bone resorption and thus aggravated bone loss. Inhibition of Nrf2 was a primary mechanism responsible for osteocytic ferroptosis.

### 2.5 Nrf2 Downregulation was Involved in RANKL Promoter Methylation by Inhibiting DNA Methyltransferase 3a (Dnmt3a)

**Figure 6:**
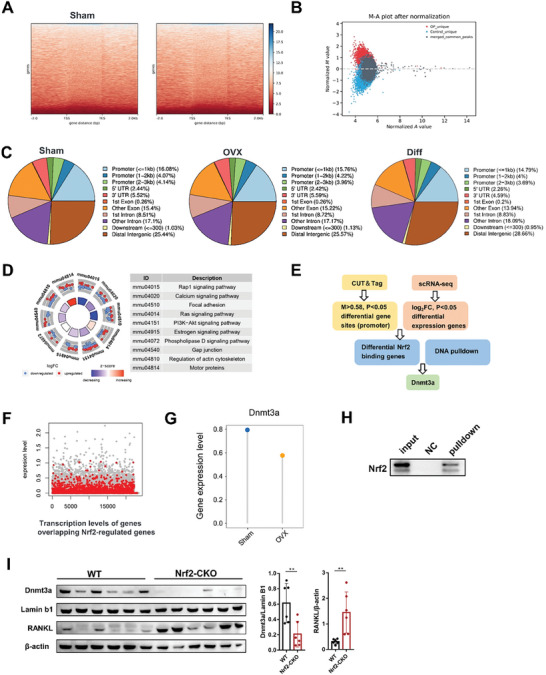
Dnmt3a in bone cells is an important downstream target regulated by Nrf2. A) Heatmaps for Nrf2 binding peaks in osteocytes from sham and OVX mice. Color depth indicates the relative number of reads, genes with similar distribution patterns are clustered together through a clustering algorithm. B) All detected peaks are plotted as a MA plot. C) Different peak distribution pie chart. D) KEGG enrichment analysis of differential Nrf2‐regulated peak in osteocytes in sham and OVX mice. E) Bioinformatics analysis filtered Dnmt3a as downstream targets of Nrf2. F) Coding genes overlapping with Nrf2‐regulated peaks were plotted (red dots) on a scatterplot showing gene transcript levels. G) Lollipop plot shows Dnmt3a expression level in osteocytes of sham and OVX mice according to the single‐cell RNA sequencing results. H) DNA pull‐down assay combined with western blotting analysis for the binding of Nrf2 to Dnmt3a gene promoter, respectively, in osteocytes using special DNA probes or negative control probe. I) Representative Western blot and quantification of the protein level of Dnmt3a and RANKL in bone tissues (femoral shaft) of the WT and Nrf2‐CKO mice (*n* = 6/group). Nrf2‐CKO: Dmp1^Cre^; Nrf2*^fl/fl^* mice. Data are represented as the mean ± SD. **P < 0.01. Two‐tailed paired t test.

Nrf2 is a pivotal regulator of cellular responses that counteract oxidative stress. Inhibition of Nrf2 triggers ferrotosis in osteocytes, thus augmenting osteoclast activity. But the effect of Nrf2 inhibition on the regulatory ability of osteocytes toward osteoclasts remains inexplicable. Osteocytes regulate the differentiation of osteoclasts predominantly through RANKL. Upregulation of RANKL was observed in Nrf2‐CKO mice using Western blotting (**Figure** **6**J). Therefore, we hypothesize that the inhibition of Nrf2 in osteocytes contributes to the enhanced expression of RANKL, potentially impacting control over osteoclast differentiation. We performed CUT&Tag using the Nrf2 antibody in osteocytes from sham and OVX mice. Heatmaps for Nrf2 binding peaks were shown in Figure 6A. M‐A plot of differentially‐bound peaks were shown in Figure 6B. Red points represent OVX‐unique peaks while blue points represent sham‐unique peaks. Genomic distributions of Nrf2‐binding sites were shown in Figure 6C. Differentially Nrf2‐binding promoter peaks were identified and subjected to KEGG analyses (Figure 6D). Differentially Nrf2‐binding promoter peaks are enriched in genes associated with estrogen signaling pathway. We investigated the potential mechanism of Nrf2 regulating RANKL by integrating CUT&Tag, scRNA‐seq, and DNA pull‐down analyses (Figure 6E). Differentially Nrf2‐binding promoter peaks based on CUT&Tag were overlapped with the differentially expressed genes by single‐cell RNA sequencing (Figure 6F). Interestingly, CUT&Tag results indicated that Nrf2 can bind to the promoter of Dnmt3a. In addition, Dnmt3a was downregulated in osteocytes from OVX mice, which was consistent with Nrf2 (Figure 6G). Subsequently, we verified the binding of Nrf2 to the Dnmt3a promoter by DNA pull‐down combined with Western blot (Figure 6H). Dual‐luciferase reporter assays results showed Nrf2 promoted Dnmt3a transcription in osteocytes (Figure S12A, Supporting Information). We also found that Nrf2 was unable to bind RANKL promoter (data not shown). The downregulation of Dnmt3a and upregulation of RANKL in osteocytes from OVX mice were also confirmed by Western blotting (Figure 6J).

**Figure 7:**
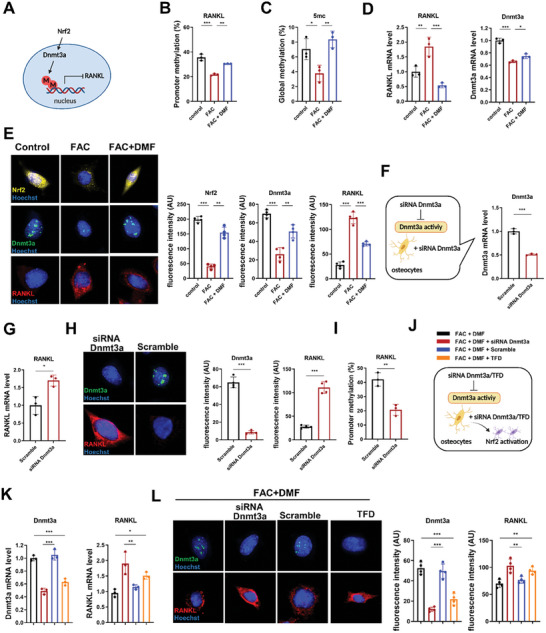
Epigenetic regulation of RANKL by Nrf2 in the ferroptotic process of osteocytes. A) Schematic diagram of Nrf2 regulation of RANKL. B) Iron overload downregulated RANKL promoter methylation in osteocytes (*n* = 3/group). C) Iron overload downregulated global DNA methylation in osteocytes (*n* = 3/group). D) Iron overload upregulated RANKL expression but downregulated Dnmt3a expression. Activation of Nrf2 by DMF (10 µm, 72 h) decreased RANKL mRNA level but increased Dnmt3a mRNA level (*n* = 3/group). E) Immunofluorescence staining and quantification of Nrf2, Dnmt3a, and RANKL in osteocytes in the control group, FAC group, and FAC+DMF group (Three randomly selected viewing fields were evaluated per sample, *n* = 3/group). Scale bar: 50 µm. F,G) siRNA Dnmt3a treatment decreased the Dnmt3a mRNA level and increased the RANKL mRNA level (*n* = 3/group). H) siRNA Dnmt3a treatment inhibited the Dnmt3a expression and enhanced the RANKL expression in osteocytes as quantified by immuno‐fluorescence (Three randomly selected viewing fields including 50–100 cells to determine average fluorescence intensity of each tissue sample, *n* = 4/group). Scale bar: 50 µm. I) siRNA Dnmt3a downregulated RANKL promoter methylation in osteocytes (*n* = 3/group). J,K) siRNA Dnmt3a and TFD (10 µm, 72 h) treatment both blocked the effect of Nrf2 on RANKL in mRNA level (*n* = 3/group). L) siRNA Dnmt3a and TFD treatment both blocked the effect of Nrf2 on RANKL in protein level as quantified by immunofluorescence (Three randomly selected viewing fields including 50–100 cells to determine average fluorescence intensity of each tissue sample, *n* = 4/group). Scale bar: 50 µm. Data are represented as the mean ± SD. *P < 0.05, **P < 0.01, ***P < 0.001, ns = not significant. Two‐tailed paired t test or ANOVA with post‐hoc Tukey–Kramer test.

DNA methylation is a type of epigenetic modification representing important mechanisms in regulating RANKL expression. Dnmt3a is involved in epigenetic gene silencing through DNA hypermethylation. Thus, we assumed that Nrf2 downregulation was involved in RANKL promoter methylation by inhibiting Dnmt3a (**Figure** **7**A). FAC treatment significantly reduced the Dnmt3a gene expression while activation of Nrf2 by DMF increased Dnmt3a gene expression in osteocytes. We then measured methylation levels of the RANKL promoter in osteocytes treated with FAC or FAC plus DMF. FAC significantly reduced methylation of the RANKL promoter and increased RANKL gene expression in osteocytes. Conversely, DMF triggered methylation of the RANKL promoter and inhibited RANKL gene expression in osteocytes. Exposed to FAC also suppressed global DNA methylation while DMF enhanced global DNA methylation in osteocytes (Figure 7B–D). Nrf2‐mediated methylation of the RANKL promoter and subsequent RANKL gene expression were further supported by a corresponding increase in Dnmt3a protein levels (Figure 7E). In addition, siRNA was employed to study the effects of Dnmt3a knockdown on RANKL expression (Figure 7F). Inhibition of Dnmt3a by siRNA rescued the deleterious effect of FAC on osteocyte RANKL at mRNA (Figure 7G) and protein levels (Figure 7H). In addition, inhibition of Dnmt3a by siRNA significantly reduced methylation of the RANKL promoter (Figure 7I). The binding of Dnmt3a to the RANKL promoter was verified by DNA pull‐down combined with Western blot (Figure S12B, Supporting Information).To further demonstrate the role of Dnmt3a in Nrf2‐mediated regulation of RANKL, we used DMF to activate Nrf2 and either siRNA or theaflavin‐3, 3′‐digallate (TFD, DNMT3a specific inhibitor),^[^ ^27^ ^]^ to inhibit Dnmt3a simultaneously (Figure 7J). Pharmacological inhibition or knockdown of Dnmt3a blocked Nrf2's negative regulation of RANKL expression, indicating that Dnmt3a‐mediated DNA methylation of RANKL promoter is a crucial factor in the regulatory axis of Nrf2 and RANKL (Figure 7K,L).

### 2.6 Nrf2 Activation Inhibited High Expression of RANKL in Osteocytes and Bone Loss of OVX Mice

**Figure 8:**
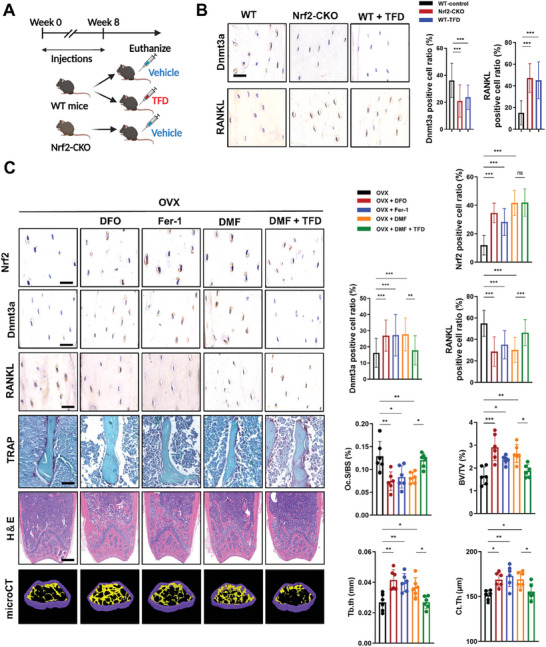
Targeting Nrf2 can effectively inhibit Dnmt3a‐mediated RANKL overexpression, thereby improving bone loss. A) Schematic showing the experimental protocol for 8 weeks of TFD injections in mice. B) IHC for Dnmt3a and Immunofluorescence staining for RANKL. Representative images are shown. SFI treatment inhibited the Dnmt3a expression while enhanced the RANKL expression in osteocytes of mice (Five randomly selected viewing fields were evaluated per section, and 6 mice were evaluated per group). Scale bar: 20 µm. C) Representative image of Nrf2, Dnmt3a, and RANKL IHC staining, TRAP staining, HE staining, and microCT 3D reconstruction are shown. Iron chelator (DFO), anti‐ferroptosis treatment (Fer‐1), and Nrf2 agonist (DMF) could to some extent suppress the expression of RANKL in osteocytes, thus inhibiting bone loss mediated by osteoclasts. TFD significantly inhibited the protective effect of DMF. Nrf2, Dnmt3a, and RANKL IHC staining Scale bar: 20 µm. HE staining Scale bar: 20 µm. TRAP staining Scale bar: 20 µm. Data are represented as the mean ± SD. *P < 0.05, **P < 0.01, ***P < 0.001. Two‐tailed paired t test or ANOVA with post‐hoc Tukey–Kramer test.

Nrf2 inhibition leaded to Dnmt3a downregulation and subsequent high expression of RANKL. We next examined whether inhibiting Dnmt3a could enhance RANKL expression in osteocytes in vivo (**Figure** **8**A). Knockout of Nrf2 significantly inhibited the Dnmt3a expression and thus increased RANKL expression in osteocytes in vivo, indicating the independent regulatory effect of Nrf2 on downstream Dnmt3a. Treated with TFD has lower Dnmt3a expression levels and higher RANKL expression levels in osteocytes compared with those in vehicle group, indicating that direct inhibition of Dnmt3a resulted in reduced DNA methylation of the RANKL promoter, thereby upregulating RANKL expression in vivo. (Figure 8B). Iron accumulation in osteocytes of OVX mice leads to downregulation of Nrf2, resulting in lipid peroxidation and finally culminating in cell ferroptosis. Lastly, we examined the effects of three anti‐ferroptotic treatments, including iron chelation therapy (DFO), Nrf2 activation (DMF) and anti‐lipid peroxidation (Fer‐1). The results showed that all three treatment methods could alleviate Nrf2 inhibition, and thus enhanced Dnmt3a expression and suppress RANKL expression in osteocytes of OVX mice. Anti‐lipid peroxidation treatment alleviated Nrf2 inhibition. This could be because Fer‐1 could quench Fe^2+^‐dependent formation of ROS and suppress ROS generated during peroxidation of polyunsaturated fatty acids (PUFAs). TRAP staining revealed that all three anti‐ferroptotic treatments inhibits the progression of osteoclastogenesis, which can be at least partially attributed to the suppression of RANKL in osteocytes. In addition, H&E staining showed that all three anti‐ferroptotic treatments could increase the density of trabecular bone in the distal femur of OVX mice. Furthermore, the µ‐CT analysis showed that all three anti‐ferroptotic treatments increased the BV/TV, Tb.Th, Tb.N, and Ct.Th, but reduced Tb.Sp in the distal femur of OVX mice. Furthermore, we activated the NRF2 pathway in DMF and additionally employed TFD to inhibit Dnmt3a. The results showed that, despite the upregulation of Nrf2 levels in osteoblasts, the expression of RANKL remained elevated due to TFD‐induced downregulation of Dnmt3a. Consequently, the protective effect of DMF against bone loss was significantly inhibited (Figure 8C).

## 3 Discussion

Osteocytes are the most numerous cell type in bone, accounting for more than 90% of total bone cells in the adult skeleton. Osteocytes are critical regulators in the maintenance of bone health.^[^ ^28^, ^29^ ^]^ Nevertheless, the role of osteocytes in postmenopausal osteoporosis is largely unknown. In our study, we found that osteocytes underwent ferroptosis in the bone of ovariectomized mice, which is primarily the result of abnormal iron metabolism in bone. Knockout of GPX4 in osteocytes aggravated osteocyte ferroptosis after OVX modeling and further exacerbated bone loss. Inhibiting osteocyte ferroptosis could effectively improve bone loss, indicating that osteocyte ferroptosis promotes bone loss. We demonstrate that osteocytic ferroptosis is associated with the regulation of osteoclast activity. RANKL expression levels were upregulated during the process of ferroptosis in osteocytes, leading to excessive activation of osteoclasts. This crosstalk between osteocytes and osteoclasts ultimately promotes bone loss. In addition, we demonstrate that Nrf2 is an important regulatory pathway for ferroptosis of osteocytes. Nrf2 protects GPX4‐knockout osteocytes from ferroptosis. The Nrf2 signaling pathway of osteocytes in mice with osteoporosis was significantly inhibited. Knockout of Nrf2 in osteocytes aggravated osteocyte ferroptosis and led to subsequent osteoclast activity. Moreover, we have confirmed that the inhibition of Nrf2 resulted in the downregulation of Dnmt3a‐mediated methylation levels of the RANKL promoter, which is an important mechanism for osteocytic ferroptosis promoting osteoclastogenesis. Therefore, we propose a new therapeutic strategy in which targeting the Nrf2 pathway improves bone loss by inhibiting ferroptosis of osteocytes and regulating the crosstalk between osteocytes and osteoclasts. Furthermore, for postmenopausal women, intervention in iron accumulation may be an effective means to prevent and alleviate osteoporosis.

Osteocytes are important mechanical stress receptors and endocrine cells in bone. Considering the large number of osteocytes, mild abnormalities in cellular function will lead to an amplification of the injury signal affecting the overall health status of the organism. The abnormal death of osteocytes was found to be related to degenerative bone disease.^[^ ^30^ ^]^ Osteoclasts and osteoblasts have been extensively studied in the occurrence and development of osteoporosis, but the role of osteocytes in osteoporosis is still unclear. Our studies add to the knowledge of the involvement of osteocytes in the development of postmenopausal osteoporosis. Recent studies have shown that the serum ferritin level of postmenopausal women is significantly increased, which was identified recently as an independent risk factor for osteoporotic fracture. At present, there are several explanations for the increase in iron levels in postmenopausal women. For example, women excrete endogenous iron mainly through menstrual blood loss, leading to a decrease in ferritin levels. After menopause, iron accumulates increasingly in the body due to the loss of this excretion pathway.^[^ ^31^, ^32^ ^]^ In addition, hepcidin, one of the most important negative regulators of iron metabolism, is a negative regulator of iron metabolism mainly secreted by the liver. Estrogen promotes the expression of calmodulin at the transcriptional level by binding to the estrogen response element in the calmodulin promoter, thus maintaining iron homeostasis. In postmenopausal women, the level of hepcidin decreases, the balance of iron metabolism is easily disrupted, and iron accumulation occurs.^[^ ^33^, ^34^ ^]^ Iron overload is an independent risk factor for osteoporosis. Iron accumulation is a change in the level of circulation. It is likely that a large number of osteocytes will be affected.

We found that serum ferritin was not increased in mice with estrogen reduction, which was inconsistent with that in human studies. However, the level of iron in bone tissues increased significantly, which attracted our attention. Previous studies also reported that serum ferritin was not significantly increased in estrogen‐reduced mice, but iron levels in the liver and brain were significantly increased.^[^ ^35^, ^36^ ^]^ We speculate that the increase in bone iron levels will have a direct impact on osteocytes. IHC showed that the ferritin level of ovariectomized mouse osteocytes increased, also confirming this hypothesis. Ferroptosis is an iron dependent, nonapoptotic, regulatory form of cell death.^[^ ^37^ ^]^ The increase in iron levels is closely related to ferroptosis. For example, brain iron levels inevitably increase in aging and degenerative diseases, which leads to a high risk of ferroptosis of a variety of cells in nervous tissue, including astrocytes, microglia and oligodendrocytes.^[^ ^38^, ^39^, ^40^, ^41^ ^]^ Here, we hypothesized and confirmed that ferroptosis occurred in ovariectomized mice osteocytes. DFO iron reduction treatment can reduce ferroptosis, which indicates that iron overload caused by estrogen reduction is the main cause of ferroptosis of osteocytes. Previous studies have shown that the apoptosis of lacunar osteocytes is associated with conditions of high bone turnover in postmenopausal women.^[^ ^42^ ^]^ Estrogen and selective estrogen receptor modulators can effectively inhibit osteocyte apoptosis.^[^ ^43^ ^]^ In addition, osteocyte necrosis could promote osteoclast‐mediated bone loss by releasing damage‐associated molecular patterns (DAMPs).^[^ ^44^ ^]^ Our research showed that ferroptosis of osteocytes is also involved in the occurrence and development of postmenopausal osteoporosis, which further confirms that abnormal death of bone cells promotes bone loss.

Many studies have shown that abnormal cell death of osteocytes is involved in the occurrence and development of orthopedic diseases, including bone marrow injury, osteoporosis, osteoarthritis and osteosarcoma.^[^ ^45^, ^46^ ^]^ Our findings support the concept that osteocytic ferroptosis plays an important role in the maintenance of bone homeostasis. In the mice after bilateral ovariectomy, after GPX4 was knocked out, severe ferroptosis occurred in the osteocytes, while the bone mass of the mice further decreased, and the bone microstructure further deteriorated, indicating that ferroptosis of the osteocytes promoted bone loss. The International Cell Death Nomenclature Committee defined ferroptosis as a form of RCD regulated by GPX4 in 2018.^[^ ^47^ ^]^ GPX4 is a key factor in the regulation of ferroptosis. However, it is worth noting that significant ferroptosis did not occur in GPX4‐deficient osteocytes in the absence of modeling, indicating the presence of other protective mechanisms. Nrf2 is one of the key regulators of the defense against the oxidative stress pathway. Nrf2 is sequestered by Keap1, promoting Nrf2 ubiquitination and degradation and thus assuring that Nrf2 basal levels remain low. Under oxidative stress, Nrf2 is stabilized and activated by dissociation from Keap1.^[^ ^48^ ^]^ Enrichr analysis results suggested that Nrf2 was an important regulatory factor in the process of osteocytic ferroptosis. We subsequently confirmed that Nrf2 is in a repressed state in iron‐deprived osteocytes by Western blotting. Nrf2, with unique pharmacological potential, has been studied in many diseases, and specific inducers have been used in clinical trials.^[^ ^49^, ^50^, ^51^ ^]^ Previous studies have also reported that GPX4 protein levels are transcriptionally regulated by the transcription factor Nrf2.^[^ ^52^, ^53^, ^54^ ^]^ Targeting Nrf2 is therefore highly likely to be a relevant established technique for the treatment of osteocyte ferroptosis. Through in vitro studies, we confirmed that agonistic Nrf2 could inhibit osteocyte ferroptosis as well as subsequent osteoclast formation. We further confirmed that osteocytes undergo severe ferroptosis in the Dmp1*^Cre+^*, Nrf2*^fl/fl^* mice. Our studies indicated that Nrf2 pathway is a key regulatory pathway for ferroptosis of osteocytes.

Postmenopausal osteoporosis is characterized by excessive bone resorption. However, osteocytes are embedded deeply in the deep bone. Although they have certain bone remodeling effects, they are only confined to the bone lacunae. The absorption of bone surface is still mainly performed by osteoclasts. Osteocytes were reported to be the main source of RANKL in mature individuals,^[^ ^14^, ^55^, ^56^ ^]^ and our study found that the expression and secretion of RANKL in ferroptotic osteocytes are increased, which suggests that osteocytes may be a key regulator rather than an executor in the process of severe bone loss caused by ferroptosis of osteocytes. In fact, many studies have also shown that osteocytes regulate the functions of various cells, including osteoclasts and osteoblasts, by transforming mechanical stress signals into biochemical signals and their powerful endocrine functions.^[^ ^57^, ^58^, ^59^, ^60^ ^]^ In addition, osteocytes communicate with other cell types, including osteoclasts, by their dendritic processes extending through a fluid‐filled canalicular network to the bone surface.^[^ ^61^ ^]^

Our study found that ferroptosis of osteocytes unregulated RANKL at both the protein and secretion levels. However, according to previous reports, membrane RANKL derived from osteocytes promotes osteoclast formation, but soluble RANKL secreted by osteocytes has little effect on osteoclast formation.^[^ ^62^, ^63^, ^64^ ^]^ Soluble RANKL contributes to physiological bone remodeling but not ovariectomy‐induced bone loss.^[^ ^65^ ^]^ Although the direct coculture model in this study cannot explain which type of RANKL is involved, our study fully shows that ferroptotic osteocytes promote bone loss by regulating the formation of osteoclasts. Based on prior literature, we speculate that the increase in membrane RANKL plays a more crucial role. In addition to RANKL, osteocytes also secrete several osteoclastogenic cytokines, including M‐CSF, IL‐1β, IL‐6 and TNF‐α,^[^ ^15^, ^66^ ^]^ which were proven to be increased in the ferroptosis of osteocytes in our study. Several studies have found that IL‐1, IL‐6, and TNF increase osteoclast formation by directly promoting the activity of osteoclast precursors. Estrogen can block the activity of these cytokines. Osteocytes in ferroptosis can abundantly secrete these cytokines, while the blocking effect diminishes due to estrogen reduction, resulting in overactivated osteoclastogenesis, which accelerates bone resorption.^[^ ^67^, ^68^, ^69^, ^70^, ^71^, ^72^ ^]^ Moreover, inflammatory factors such as TNF‐a can directly enhance osteocyte RANKL expression and promote osteoclast formation.^[^ ^73^ ^]^ Ferroptotic osteocytes release inflammatory factors such as IL‐1β and TNF‐a, which may act on the osteocytes themselves to further induce high RANKL expression in osteocytes, forming an amplifying effect. After the ferroptosis of osteocytes was inhibited by the activation of the Nrf2 pathway, the activation effect of osteocytes on osteoclasts was prevented, indicating that targeting the Nrf2 signaling axis to inhibit ferroptosis of osteocytes can effectively prevent osteoclast‐mediated bone destruction activities.

A high expression of RANKL in osteocytes is a key factor in osteoclast activation. But the specific mechanisms of the high expression of RANKL in pathological states are still unclear. DNA methylation is the most well‐characterized epigenetic modification which has been shown to be associated with the upregulation of RANKL in osteoblasts and blood samples.^[^ ^74^, ^75^ ^]^ We found that significantly decreased DNA methylation in RANKL promoter in osteocytes is an important reason for the upregulation of RANKL. Activation of Nrf2 can inhibit DNA methylation of RANKL promoter and thus suppressed the expression of RANKL, suggesting the existence of a novel mechanism by which Nrf2 can regulate RANKL. Dnmt3a, a DNA methyltransferase, acts as de novo methyltransferase for modification of unmethylated DNA.^[^ ^76^ ^]^ Dnmt3a^−/−^ myeloid cells, especially macrophages, promote osteoclast differentiation via secreting molecules that increase osteoclast differentiation.^[^ ^77^ ^]^ Dnmt3a was downregulated in osteocytes from OVX mice, which may also affect the expression and secretion of osteoclastogenic cytokines. In our study, Dnmt3a was identified as a downstream target of Nrf2 in our study. Nrf2 can regulate Dnmt3a expression levels by binding to its promoter and directly regulating its transcription. Nrf2 activators can significantly upregulate Dnmt3a expression levels, whereas inhibiting Nrf2 expression leads to a corresponding downregulation of Dnmt3a. Our results showed that Dnmt3a directly participates in RANKL promoter methylation. Knocking down its expression can lead to RANKL upregulation, indicating that Dnmt3a acts as an intermediate regulator in Nrf2's modulation of RANKL. DMF treatment significantly enhanced Dnmt3a expression, inhibited RANKL expression in osteocytes and suppressed osteoclast‐mediated bone resorption and bone loss, providing favorable evidence to support the Nrf2/Dnmt3a/RANKL axis as an important mechanism in osteocytic ferroptosis‐mediated osteoclastogenesis.

Previous studies on postmenopausal osteoporosis have mainly focused on the direct effect of estrogen reduction on the formation of osteoclasts. However, the role of osteocytes in postmenopausal osteoporosis still needs to be clarified. Recent studies have shown that damage to the function of osteocytes can lead to a variety of bone diseases,^[^ ^17^, ^18^, ^78^ ^]^ but the changes experienced by osteocytes in postmenopausal osteoporosis patients have not been determined. In this study, we determined that osteocytes in postmenopausal osteoporosis experienced ferroptosis and that ferroptotic osteocytes affected the process of bone loss by regulating osteoclast‐mediated bone resorption. In addition, we confirmed that Nrf2 acts as a key regulatory pathway to affect bone loss mediated by osteocyte ferroptosis. Our study demonstrated that Nrf2/Dnmt3a/RANKL axis in osteocyte is a new mechanism of osteoporosis. Similarly, based on our findings, we believe that this pathway can be used as a therapeutic target to improve the progression of postmenopausal osteoporosis.

However, our research also has some limitations. First, the hormone changes in the postmenopausal body are complex, and the ferroptosis of osteocytes cannot be completely explained by iron accumulation. In addition to the Nrf2 pathway, other important regulatory pathways need to be further clarified. Finally, we need to determine the more specific mechanisms that explain the increased expression of RANKL in osteocytes with ferroptosis.

## 4 Experimental Section

### Cell Culture Studies

Ocy454 cells, a murine osteocyte line, were kindly provided by Prof. Paola Divieti Pajevic (Boston University, Henry M. Goldman School of Dental Medicine). Ocy454 cells were differentiated in α‐minimum essential medium (α‐MEM, Gibco; Thermo Fisher Scientific, Inc.) containing 10% fetal bovine serum (FBS, Gibco) with 5% CO2 at 37 °C for 10 days before subsequent experiments. MC3T3‐E1 cells were purchased from The Cell Bank of Type Culture Collection of the Chinese Academy of Sciences and cultured in α‐MEM containing 10% FBS. Primary bone marrow‐derived macrophages (BMMs) were extracted from the femurs and tibias of 4‐week‐old female C57BL/6 mice (Shanghai Slake Laboratory Animal Co., Ltd., Shanghai, China) as described previously.^[^ ^79^ ^]^ BMMs were cultured in α‐MEM containing 10% FBS and 30 ng ml^−1^ M‐CSF (R&D Systems, Minneapolis, MN, USA) with 5% CO2 at 37 °C.

### Measurement of Cell Viability

Ocy454 cells were seeded on 96‐well plates at a density of 5 × 10^3^ cells per well. During pretreatment, the cells (50000 cells/well) were pretreated with 25 nm Lip‐1, 10 µm Fer‐1, 20 µm Z‐VAD‐FMK or 50 µm Nec‐1 at 37 °C for 24 h. Cells were then treated with 150 µm FAC for 72 h. Cell viability was measured using the CCK‐8 kit (Beyotime, Nanjing, China). Briefly, cells were washed with phosphate‐buffered saline (PBS, Gibco) and cultured with 10% CCK‐8 solution for 1 h at 37 °C. Optical density was read at 450 nm using a microplate reader (Epoch; BioTek Instruments, Inc., USA).

### ROS Generation Detection

ROS levels were assessed using 2′,7′‐dichlorofluorescein diacetate (DCFH‐DA, Sigma) staining. Briefly, cells were collected and stained with 10 µm DCFH‐DA for 30 min. Flow cytometry analysis was performed using a BD Accuri C6 plus flow cytometer (BD Biosciences, Vianen, The Netherlands).

### Measurement of Lipid Peroxidation In Vitro and In Vivo

Lipid peroxidation was measured using C11‐BODIPY 581/591 (Invitrogen) staining as described previously.^[^ ^80^ ^]^ Briefly, cells and frozen sections of mouse femurs were incubated with 2 µm C11‐BODIPY 581/591 for 30 min followed by Hoechst 33 342 for 5 min. Fluorescent images were captured using a confocal microscope (Olympus, FV3000, Germany). In addition, cells were collected, stained with 2 µm C11‐BODIPY 581/591 for 30 min and analyzed by flow cytometry.

### FerroOrange Stainging

A FerroOrange probe (Dojindo, Japan) was used to detect intracellular Fe^2+^. For in vitro studies, after the indicated treatments, osteocytes were washed with PBS solution and treated with FerroOrange working solution (1 µm) for 30 min. Finally, the fluorescence was detected by flow cytometry. For in vivo studies, osteocytes were first isolated from mouse femurs as previously reported.^[^ ^81^ ^]^ Osteocytes were labelled with two flow type antibodies anti‐SPARC and anti‐ Podoplanin according to previous research.^[^ ^81^ ^]^ The cells were labeled by the antibodies, stained with FerroOrange and then sent to the flow cytometry.

### Transmission Electron Microscopy

Cells or mouse femurs were fixed with 2% glutaraldehyde for 24 h and postfixed in 1% osmic acid at 37 °C for 1 h. Samples were dehydrated with a graded series of ethanol and embedded in Epon812 resin. Ultrathin sections were stained with uranyl acetate and lead citrate and observed with a Hitachi H‐7500 transmission electron microscope (Hitachi).

### Western Blotting

Cell and tissue proteins were extracted using RIPA buffer containing cocktail. Nuclear protein was extracted by using a nuclear protein extraction kit (Beyotime). Protein concentrations were quantified by BCA assay (Thermo Fisher Scientific). Western blotting was performed as previously described. Briefly, equal amounts of protein were resolved by SDS/PAGE in each experiment and then transferred to polyvinylidene difluoride (PVDF) membranes (Millipore, ISEQ00010). Membranes were blocked with 5% nonfat dried milk and incubated with the following specific primary antibody overnight at 4 °C: GPX4 (1:2000; 14432‐1‐AP, Proteintech, Wuhan, China), SLC7A11 (1:2000; 26864‐1‐AP, Proteintech), Ferritin Light Chain (FTL, 1:2000; ab69090, Abcam), Ferritin Heavy Chain (FTH, 1:1000; ab183781, Abcam), Nrf2 (1:1000, 16396‐1‐AP, Proteintech), Keap1 (1:1000, 60027‐1‐Ig, Proteintech), heme oxygenase‐1 (HO‐1, 1:2000; 27282‐1‐AP, Proteintech), NQO‐1 (1:2000, 11451‐1‐AP, Proteintech), RANKL (1:2000; 23408‐1‐AP, Proteintech), Dnmt3a (1:2000, ab188470, Abcam), Lamin B1 (1:2000, 12987‐1‐AP, Proteintech) and β‐actin (1:2000; #4970, Cell Signaling Technology). The membranes were then incubated with goat anti‐rabbit IgG (H+L) secondary antibody for 2 h at room temperature. Signals of target proteins were visualized using enhanced chemiluminescence on an imaging system (Tanon, Shanghai, China). The gray values of Western blot images were quantified by ImageJ software (version 1.8.0; National Institutes of Health, Bethesda, MA, USA). Relative protein levels were normalized to those of β‐actin or lamin B.

### Establishment of Coculture Systems

Osteocyte–osteoclast coculture was performed as previously described.^[^ ^82^ ^]^ After the different conditional treatments, Ocy454 cells were seeded at 500 cells per well on 96‐well plates or 1 × 10^4^ cells per well on 6‐well plates for 24 h. BMMs were subsequently seeded at 5 × 10^3^ cells per well or 1 × 10^5^ cells per well on 6‐well plates to establish the direct coculture system. These cells were cocultured in α‐MEM containing 10% FBS and 30 ng ml^−1^ M‐CSF for 7 days. For osteocyte–osteoblast coculture, Ocy454 cells were seeded at 500 cells per well on 96‐well plates for 24 h, and MC3T3‐E1 cells were then seeded at 5 × 10^3^ cells per well. These cells were cocultured in osteogenic induction medium with 50 µg ml^−1^ vitamin C (Sigma), 10 mm ß‐glycerolphosphate (Sigma), and 0.1 µm dexamethasone (Sigma).

### TRAP Staining

After 7 days of osteocyte–osteoclast coculture, cells were then fixed with 4% paraformaldehyde (PFA) for TRAP staining using a TRAP assay kit (TaKaRa Bio, Inc., Shiga, Japan) according to the manufacturer's instructions. Cells were observed under a microscope. TRAP‐positive multinucleated cells with ≥3 nuclei were considered osteoclasts.

### Immunofluorescence Assay

Cells were fixed, blocked with QuickBlock Blocking Buffer (Beyotime), incubated with anti‐RANKL antibody (1:200; 23408‐1‐AP, Proteintech), anti‐Dnmt3a antibody (1:200; ab188470, Abcam), and then stained with labeling fluorescent secondary antibodies. Incubation with Hoechst 33 342 for 5 min was performed to visualize cell nuclei. The cells were observed under a confocal microscope.

### Bone Resorption Pit Assessment

For bone resorption assessment, BMMs and Ocy454 cells were seeded on bovine bone slides for coculture. After 7 days of treatment, cells were removed from bone slides using sodium hypochlorite solution. Bone resorption pits were visualized using scanning electron microscopy (SEM). Bone resorption areas were quantified using ImageJ software (version 1.8.0; National Institutes of Health, Bethesda, MA, USA).

### ALP and ARS Staining

ALP staining was performed using a BCIP/NBT alkaline phosphatase color kit (Beyotime). After 7 days of osteogenic induction, cells were fixed with 4% paraformaldehyde and stained with ALP according to the manufacturer's instructions. The mineralization capacity of osteoblasts was evaluated by ARS staining. After 21 days of osteogenic induction, the cells were washed with PBS, fixed in 4% paraformaldehyde solution and stained with ARS staining solution for 30 min. The BMSCs were observed under an Olympus fluorescence microscope at a magnification of 40×. The positive area of ALP staining and the mineralized modules were quantified using ImageJ software.

### Methylation‐Specific PCR

A total of 200 ng of genomic DNA underwent bisulfite conversion using the EZ DNA Methylation Kit (Zymo Research, Irvine, CA), followed by methylation‐specific PCR (MSPCR) with primers specifically designed for methylated DNA of the RANKL promoter. The primer sequences were as follows: left primer, 5′‐ AAAGATGGATGTTTGTTGATATTTT‐3′ and right primer, 5′‐ ACCAAATTCTTTCCATATACTACCAC‐3′. To calculate the percentage of DNA methylation in the samples, fully methylated control DNA (Zymo Research, Irvine, CA) was used as a reference.

### Global DNA Methylation Assessment

The levels of 5‐methylcytosine (5mC) in DNA were assessed using the 5mC DNA ELISA Kit (Zymo Research, Irvine, CA). Two hundred nanograms DNA was utilized, and the percentage of 5mC in the samples was determined, normalized to the total DNA amount, and compared to the provided standard curve in the kit.

### RT‐qPCR

Total RNA was extracted from osteocytes using TRIZOL reagent (Invitrogen, Carlsbad, CA, USA). Single‐stranded cDNA was synthesized from extracted RNA using a reverse transcription master mix (Takara, Shiga, Japan). Quantitative RT‐PCR was performed using the SYBR green qPCR master mix (EZBioscience, Roseville, USA) on a LightCycler 480 II RT‐PCR instrument (Roche Diagnostics, Basel, Switzerland). GAPDH expression was used to normalize the Ct values. Gene expression was assessed using the 2−ΔΔCt method

### Luciferase Reporter Assays

Dnmt3a promoter‐reporter plasmid and Nrf2 overexpression plasmid (pcDNA3.1‐N‐HA) were constructed by (Nanjing, *China*). Dnmt3a promoter‐luciferase reporter plasmid was constructed by inserting the amplified ∼2 Kb sized Dnmt3a promoter into the modified pGL3‐Basic. For the luciferase reporter assay, Ocy454 cells were transfected with the constructed plasmids plasmids a using a CTX‐1500A EX Electroporator (Celetrix Biotechnologies, Manassas, VA, United States). Cells were co‐transfected with Renilla (Promega) as a normalization control. 48 h after transfection, cells were harvested and lysed by EBC buffer. Luciferase assay was performed using the Dual‐Luciferase Reporter Assay System (Promega, E1960).

### Mice

GPX4 flox (Jax 02 7964) and Nfe2l2 flox (Jax 02 5433) mice were obtained from the Jackson Laboratory (USA). H11‐Dmp1‐iCre mice (strain No. T004830) were purchased from GemPharmatech (Nanjing, China). GPX4^fl/f^ mice were crossed with Dmp1*^Cre+^* mice to generate GPX4OcyKO (Dmp1*^Cre+^*; GPX4 *^fl/fl^*) and control (Dmp1*^Cre+^*; GPX4^+/+^) mice. Nef2l2 *^fl/fl^* mice were crossed with Dmp1*^Cre+^* mice to generate GPX4OcyKO (Dmp1*^Cre+^*; Nef2l2*^fl/fl^*) and control (Dmp1 *^Cre+^*; Nef2l2^+/+^) mice. Littermate controls were used for all studies. All animal experiments were approved by the Animal Research Committee of the Shanghai Sixth People's Hospital. All animals were housed in Shanghai Sixth People's Hospital.

### OVX Mouse Model Establishment and BMD Measurement

Osteoporosis was induced through ovariectomy, as previously described.^[^ ^79^, ^83^ ^]^ All animals were adaptively fed for 1 week prior to use. Eight‐week‐old female mice were anesthetized by isoflurane inhalation. The backs of the mice were shaved and disinfected using iodine solution. The peritoneal cavity was then opened, a ligature was placed, and the ovary was removed. The incision in the abdominal wall was closed with sutures. For drug treatments, mice received DFO (200 mg k^−1^g, every three days), Fer‐1 (5 mg k^−1^g, every three days), ML385 (30 mg k^−1^g, daily), DMF (10 mg k^−1^g, daily), or TFD (5 mg k^−1^g, daily) via intraperitoneal injection for 8 weeks. Drugs were administered one week after surgery in OVX mice. The lumbar spine BMD, femur BMD and total BMD (skull excluded) of mice were measured with dual X‐ray absorptiometry (DXA, iNSiGHT VET DXA; OsteoSys, Seoul, Korea) after 8 weeks of treatment.

### Enzyme‐Linked Immunosorbent Assay (ELISA)

The serum ferritin and C‐terminal telopeptide of type I collagen (CTX‐1) levels and in vitro RANKL, IL‐1β, IL‐6, TNF‐α, and M‐CSF from Ocy454 cells were determined using corresponding ELISA kits (Kenuodi Biotechnology Co., Ltd., Quanzhou, China) following the manufacturer's instructions. The concentrations of ferritin, CTX‐1, RANKL, IL‐1β, IL‐6, TNF‐α, and M‐CSF were calculated from standard curves.

### Bone Iron Measurement

Femurs were harvested, and the soft tissue was carefully removed. The metaphysis and diaphysis of femurs were separated. Bone marrow was removed by flushing with PBS. A total of 0.1 g of cortical bone of the femoral shaft, 5 ml of aqua regia, and 0.3 ml of hydrofluoric acid were added to 50 ml of polytetrafluoroethylene. The mixture was heated to dryness on a heating plate before being added to 5 ml of nitric acid (10%) solution. The samples were evaporated to reduce the volume to 1 mL and transferred to a volumetric flask (25 mL). The final volume of the samples was made up to 25 mL using nitric acid (10%). The amount of iron in the samples and standards was subsequently determined by inductively coupled plasma‒optical emission spectrometry (ICP‒OES, Avio 200, Perkin Elmer, Avio‐200, PerkinElmer, USA).

### Microcomputed Tomography (µ‐CT) Analysis

The mouse femurs were harvested and scanned using a SCANCO 50 (Switzerland). Representative 3D images were generated using CTVol software (SkyScan). Regions of interest were analyzed 300 slices (1.8 mm) above the growth plate of the distal femur. Bone volume per tissue volume (BV/TV), trabecular number (Tb.N), trabecular separation (Tb.Sp), trabecular thickness (Tb.Th), and cortical thickness (Ct.Th) were generated to evaluate trabecular morphometry using CTan software (Skyscan).

### Histology and Immunohistochemistry

Femurs were collected and fixed in 4% PFA overnight. The femurs were decalcified and embedded in paraffin. The slices were stained with hematoxylin and eosin (H&E) and Masson's trichrome staining. For IHC analysis, 4‐µm‐thick tissue slices were mounted on slides, baked, dewaxed with xylene and hydrated with a gradient ethanol series (100%, 95%, 80%, and 75% for 5 min each). Antigen repair was conducted using the microwave method. Endogenous peroxidase activity was blocked using 3% H2O2 for 10 min. The samples were incubated with 5% bovine serum albumin (Sigma‐Aldrich) for 30 min at room temperature to eliminate nonspecific staining. Samples were incubated with primary antibodies at 4 °C overnight. The samples were incubated with the secondary biotinylated antibody (Proteintech) for 30 min at 37 °C. Finally, the samples were stained with 3,3′‐diaminobenzidine tetrahydrochloride at room temperature for 25 s and counterstained with hematoxylin at room temperature for 5 min. The slides were scanned using a pathological section scanner (Leica SDPTOP HS6).

### Calcein Double Labeling

Calcein double labeling was performed to evaluate the extent of new bone formation in mice. All mice were administered calcein (20 mg k^−1^g, Sigma‐Aldrich) at 10 and 2 days before the animals were sacrificed via subcutaneous injection. The mineralizing surface/bone surface (MS/BS), bone formation rate/bone surface (BFR/BS), and mineral apposition rate (MAR) were analyzed using ImageJ.

### Single‐Cell RNA Sequencing

Single‐cell suspensions were prepared from bone tissue of mice femur samples as reported previously.^[^ ^84^ ^]^ Freshly prepared cell suspensions were performed immediately according to the manufacturer's protocol of 10 X Chromium 3′ v3 kit (10x Genomics, Pleasanton, CA). Library was prepared and sequencing was performed on a NovaSeq 6000 platform (Illumina, Inc., San Diego, CA) in GENERGY BIO (Shanghai, China).

### Read Alignment and Quality Control

Raw reads obtained from scRNA‐seq experiments were aligned to the mouse genome (mm10) using the CellRanger pipeline (cellranger‐7.0.1, 10X Genomics). To obtain a high‐quality data, the following filtering process was performed. 1) Cells with detected genes less than 500 and more than 7500 were removed for further analysis. 2) Cells with %Mitochondrial genes greater than 10% were removed to rule out apoptotic cells. After that, R package “DoubletFinder” (version 2.0.3) was applied to predict and remove potential doublets within each sample. Finally, there were 23491 genes and 51690 cells left for downstream analysis.

### Dimension Reduction and Clustering Analysis

After quality control, the preprocessed gene expression data of 51690 cells were analyzed by the Seurat package (version 4.2.0) and the following steps were performed in order: data normalization and transformation, highly variable gene selection, principal component analysis (PCA) and clustering. The count matrix was first normalized and transformed using function SCTransform. The top 3000 highly variable genes were then obtained by FindVariableGenes with the default variance stabilizing process. Top 30 principal components were used for clustering and the resolution of FindClusters was set to 0.4. To eliminate the batch effect, harmony algorithm was performed in Harmony R package before clustering analysis. Uniform Manifold Approximation and Projection (UMAP) was used for the final dimension reduction and visualization.

Marker genes were identified by comparing the mean expression of each gene in one cell type against mean of average expression in all other cell types by using function FindAllMarkers with the parameter method = MAST in Seurat package. Top marker genes were selected according to the adjusted p‐value and log2 fold change (log2 FC) within each cluster and cell clusters were determined based on previously known cell type marker expression.

### Differential‐Expression Analysis

Within each cluster, DEGs was detected between sham and OVX conditions by using “FindMarkers” function with parameter “test.use = MAST”. Then threshold p < 0.05, Fold change > = 1.2 were set to filter DEGs and obtained OVX up‐ and down‐regulated genes compared to Sham for each cluster. The DEGs functional enrichment analysis based on GO and KEGG was applied by an R package ClusterProfile (version 4.6.1) using a hypergeometric test and corrected for multiple hypothesis by FDR.

### Pseudotime Trajectory Analysis

Pseudotime trajectory inference of osteoblast and osteocyte population was carried out using the workflow suggested in the Monocle2 tutorial. In brief, the newCellDataSet function was applied to generate a data structure for trajectory analysis. Then, after normalization, reduceDimension and orderCells were used to reduce the dimensionality and order cells in the pseudotime trajectory. The genes used for pseudotime trajectory analysis came from the differentialGeneTest function (FDR adjusted p‐value < 0.01).

### Cell–Cell Communication Analysis

To further investigate the intercellular communication changes between Sham and OVX, ligand‐receptor analysis was performed using CellChat (version 1.6.1) according to tutorials provided on GitHub (https://htmlpreview.github.io/?github.com ^−1^ sqjin/CellChat/blob/master/tutorial/Comparison_analysis_of_multiple_datasets.html). Briefly, for Sham and OVX samples independently, identifyOverExpressedGenes and identifyOverExpressedInteractions were sequentially run with default parameters to find the ligand‐receptor gene combinations overexpressed in these cell types. Next, computeCommunProb was run followed by computeCommunProbPathway, netAnalysis_computeCentrality, and aggregateNet with default parameters to find the ligand‐receptor pathways present. Lastly, the Sham and OVX objects were merged and ran computeNetSimilarityPairwise with type “functional”. Finally, the compareInteractions and netVisual_bubble were used to visualize the results.

### CUT&Tag Analysis

Osteocytes were collected for CUT&Tag analysis. CUT&Tag analysis was performed according to published methods.^[^ ^85^ ^]^ The HyperactiveTM In Situ ChIP Library Prep Kit for Illumina (Catalog number TD901‐TD902, Vazyme Biotech Co., Ltd) was used to construct the CUT&Tag library. The experimental procedures were conducted according to the manufacturer's instructions. Anti‐Nrf2 antibody (ab62352, Abcam) was used in CUT&Tag analysis. The quality of libraries was determined using Agilent 2100 Bioanalyzer. Data were generated using NovaSeq 6000 in paired‐end 150 bp mode. CUT&Tag was analyzed following the methods available at https://yezhengstat.github.io/CUTTag_tutorial/. Briefly, quality of CUT&Tag data sets was assessed using the FACTQC tool (v.0.11.2). CUT&Tag raw reads were trimmed using TrimGalore(v.0.6.4). The trimmed reads were aligned to the mouse reference genome GRCm39 (mm39) using Bowtie2. ChIPseeker (v1.8.0) R package was used for peak annotations. Differential analysis was performed using the Manorm software. A regression model was established for M‐value and A‐value, and subsequently corrected the M‐value and A‐value to eliminate the effects of sequencing bias. The p‐value was calculated based on the Bayesian model. Finally, differential peaks were determined based on the M‐value and p‐value, with a threshold of p‐value<0.05 and |M‐value|>0.58 for filtering.

### DNA Pull Down

DNA pull‐down assay was performed as reported previously.^[^ ^86^ ^]^ Briefly, probe targeted with Dnmt3a promoter was designed and labeled with desthiobiotin. Nuclear proteins of osteocytes were extracted, incubated with the DNA probe and then bound with streptavidin‐containing magnetic beads. After washing with PBS for three times, the eluates were subjected to western blotting analysis for the target proteins.

### Code Availability

No customized code was used for data analyses in this study. Any additional information required to reanalyze the data reported in this paper is available from the lead contact upon request.

### Statistical Analysis

All experiments were performed at least three times. Data were expressed as the means of triplicate biological repeats within a representative experiment plus/minus standard error. Statistical analyses between two groups were performed using an unpaired two‐tailed Student's t test. When more than two experimental groups were present, ANOVA followed by the post hoc Tukey–Kramer test was performed. P values <0.05 were considered to be significant. The variation between groups was similar in all cases.

## Conflict of Interest

The authors declare no conflict of interest.

## Supporting information

Supporting Information

## Data Availability

Research data are not shared.
