# Supplementary material for: Ferroptosis in Osteocytes as a Target for Protection Against Postmenopausal Osteoporosis
Source: Adv Sci (Weinh). 2024 Jan 17;11(12):2307388. doi: 10.1002/advs.202307388 (PMC10966575; doi:10.1002/advs.202307388)
Supplement: Supplementary file 1 — Supporting Information [file ADVS-11-2307388-s001.pdf]

ADVANCED SCIENCE
Open Access

Supporting Information

for *Adv. Sci.*, DOI 10.1002/advs.202307388

Ferroptosis in Osteocytes as a Target for Protection Against Postmenopausal Osteoporosis

Zengxin Jiang, Guobin Qi, Xuecheng He, Yifan Yu, Yuting Cao, Changqing Zhang, Weiguo Zou* and Hengfeng Yuan**

Supporting Information

Ferroptosis in osteocytes as a target for protection against postmenopausal osteoporosis

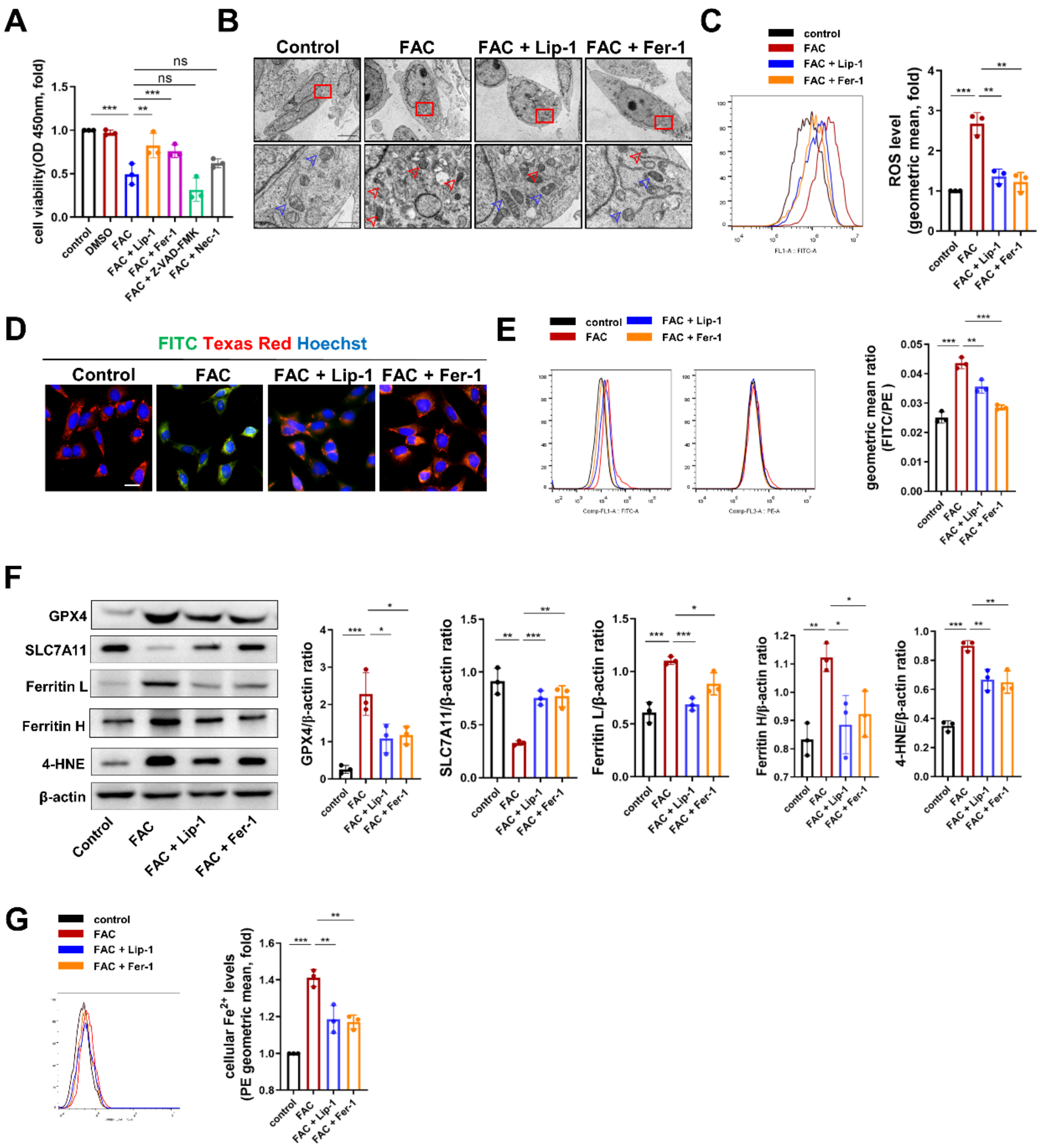

Figure S1. Ferroptosis was the predominant form of cell death in Ocy454 cells in a high-iron environment. (A) CCK-8 assays showed that inhibition of ferroptosis (25 nM Lip-1 and 10 μM Fer-1) was more effective in improving cellular viability than inhibition of apoptosis (20 μM Z-VAD-FMK) and necroptosis (50 μM Nec-1) in FAC-treated Ocy454 cells (n = 3). (B) Representative images of mitochondria in Ocy454 cells (detected by transmission electron microscopy, scale bar, upper 5 μm, lower 1 μm; red dox denotes the region shown at higher magnification. blue arrow, normal mitochondria; red arrow, abnormal mitochondria). (C) ROS levels in Ocy454 cells were determined by flow cytometry and quantitation (n = 3). (D) Ocy454 cells were stained with C11-Bodipy 581/591, observed under a confocal fluorescence microscope and (E) quantified using flow cytometry to assess lipid peroxidation. (scale bar, 100 μm, n = 3, red: normal lipids; green: oxidized lipids; blue: cell nuclei). (F) Representative Western blot and quantification of the protein levels of GPX4, SLC7A11, Ferritin Light Chain, Ferritin Heavy Chain and 4-HNE (n = 3). (G) Fe^{2+}

levels in Ocy454 cells were determined by FerroOrange staining and quantitation (n = 3). Data are represented as the mean ± SD. *P < 0.05, **P < 0.01, ***P < 0.001, ns = not significant.ANOVA with post-hoc Tukey–Kramer test.

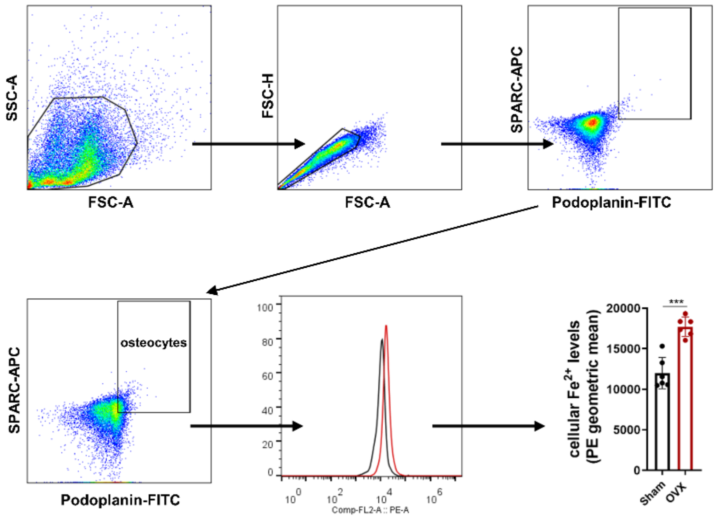

Figure S2. Obvious increase of Fe^{2+} was observed in osteocytes of OVX mice. The osteocytes were marked by Podoplanin-FITC and SPARC-APC. The gating strategy was shown. Fe^{2+} levels significantly increased in osteocytes of OVX mice (n = 6). Data are represented as the mean ± SD. *P < 0.05, ***P < 0.001. Two-tailed Student’s t test.

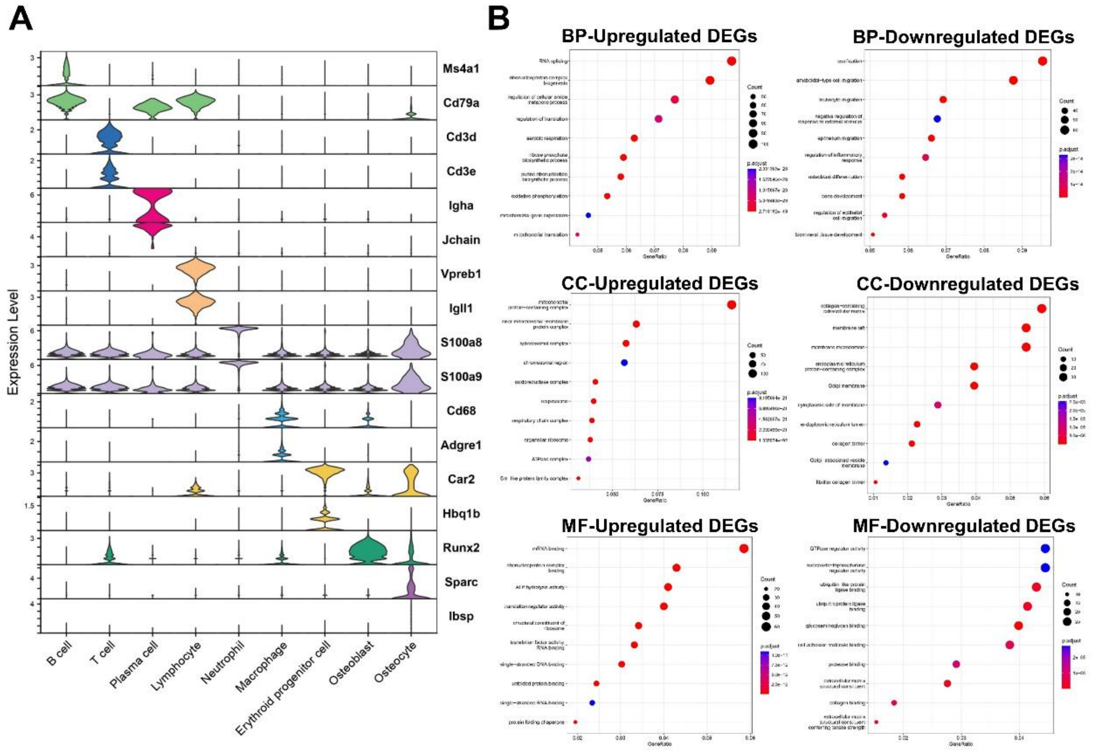

Figure S3. Violin plots of specific genes for each cluster and GO enrichment analysis of DEGs. (A) Violin plots plot showing the expression of specific signatures in identified cell types. (B) GO enrichment analysis of differentially expressed genes in osteocytes in sham and OVX mice.

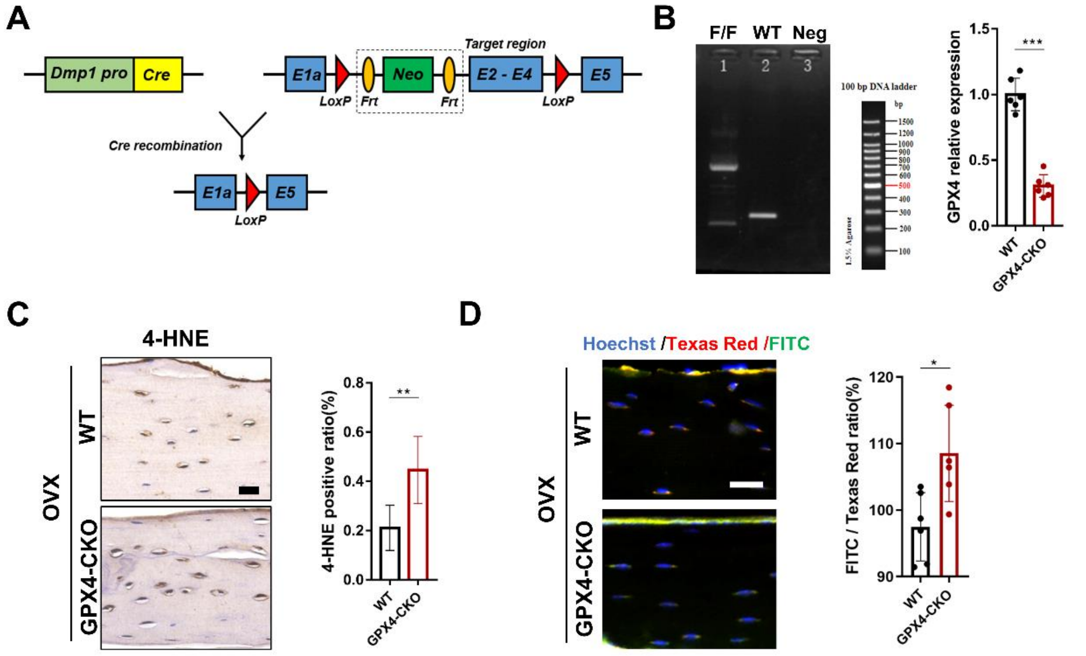

Figure S4. GPX4-deficient osteocytes exhibited ferroptotic manifestations. (A) Dmp1^{Cre}; GPX4$^{fl/fl}$ mouse construction strategy. (B) Genotyping of the GPX4$^{fl/fl}$ and WT mice. (B) qPCR analysis of GPX4 expression in the bone tissue (femoral shaft) of the WT and Dmp1^{Cre}; GPX4$^{fl/fl}$ mice (n = 6). (C) Representative images of IHC staining for detecting 4-HNE and quantification (scale bar, 20 μm). Five randomly selected viewing fields were evaluated per section, and 6 mice were evaluated per group. (D) Lipid peroxidation determined by C11-Bodipy staining from WT and GPX4-CKO mice (n = 6, scale bar, 20 μm, red: normal lipids; green: oxidized lipids; blue: cell nuclei). GPX4-CKO: Dmp1^{Cre}; Gpx4$^{fl/fl}$ mice. Data are represented as the mean ± SD. *P < 0.05; **P < 0.01; ***P < 0.001.Two-tailed Student's t test.

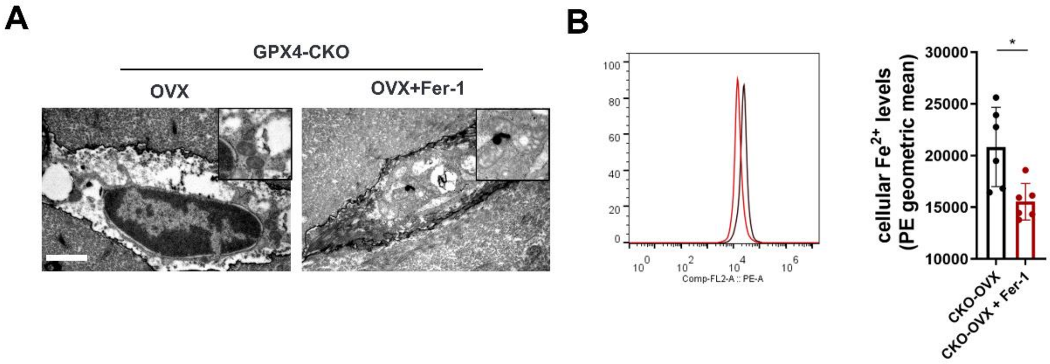

Figure S5. Fer-1 protected osteocytes in GPX4-CKO-OVX mice from ferroptosis. (A) TEM was used to observe the morphological changes in mitochondria of osteocytes in the femur of GPX4-CKO-OVX mice treated with and without Fer-1. Scale bar: 2 μm. (B) Fe^{2+} levels in osteocytes of GPX4-CKO-OVX mice treated with and without Fer-1 were determined by FerroOrange staining and quantitation (n = 6). GPX4-CKO: Dmp1^{Cre}; Gpx4$^{fl/fl}$ mice. Data are represented as the mean ± SD. *$P < 0.05$. Two-tailed Student’s t test.

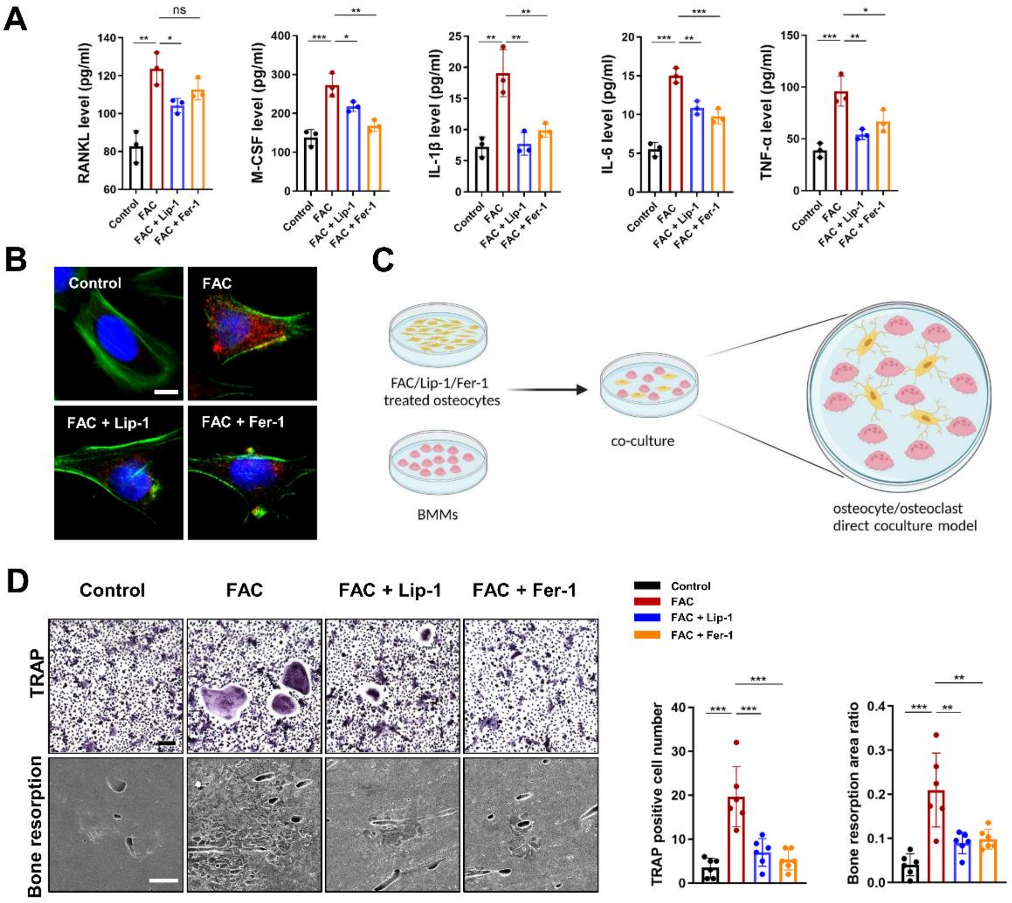

Figure S6. Ferroptosis of osteocytes promoted the activation of osteoclasts. (A) The secretion levels of RANKL, M-CSF, IL-1β, IL-6, and TNF-α in Ocy454 cells in different groups (n = 3). (B) Representative pictures of RANKL immunofluorescence in Ocy454 cells (scale bar, 10 μm, red: RANKL; green: phalloidin; blue: cell nuclei). (C) Schematic of the osteocyte/osteoclast direct coculture experiments. (D) Representative pictures of TRAP staining (scale bar, 100 μm) of BMMs and bone resorption pits (examined by scanning electron microscopy, scale bar, 100 μm). Quantification of TRAP-positive multinucleated (≥3 nuclei) cell number per well (n = 3) and bone resorption pit area ratio (n = 3). Data are represented as the mean ± SD. *$P < 0.05$, **$P < 0.01$, ***$P < 0.001$, ns = not significant. ANOVA with post-hoc Tukey–Kramer test.

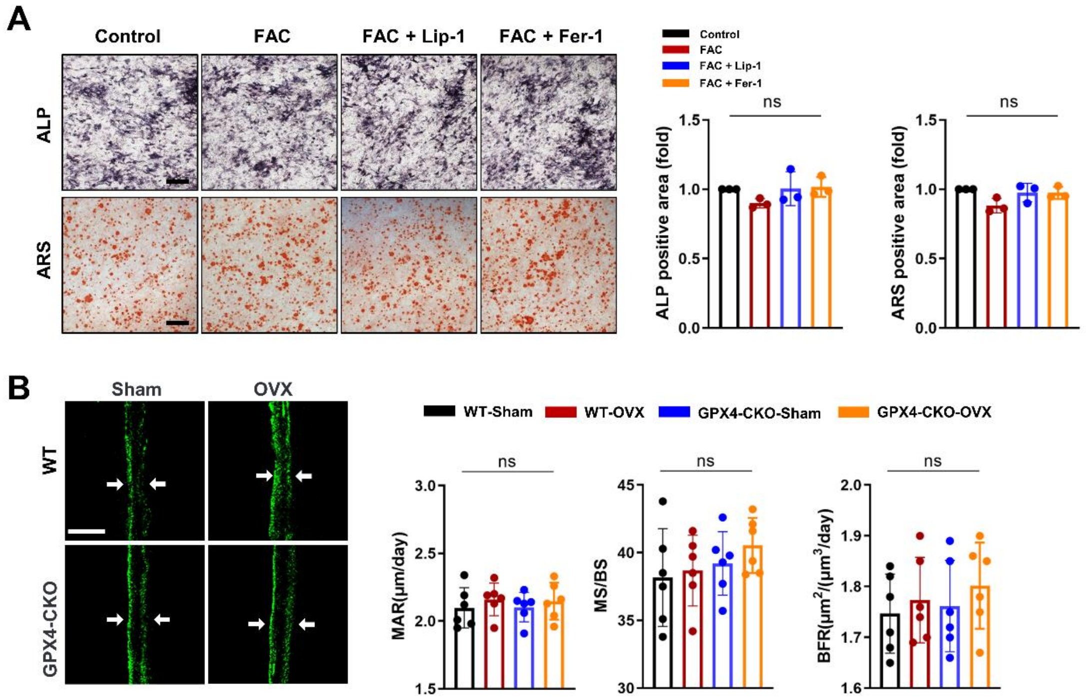

Figure S7. Ferroptosis of osteocytes did not significantly inhibit osteoblast-mediated bone formation. (A) Representative ALP and ARS staining images (scale bar, 100 μm). Quantitative analysis of the ALP-positive area and calcium deposition normalized to the control group (n = 3). (B) Representative fluorescence images of calcein double labeling in femurs of mice (scale bar, 20 μm). Mineral apposition rate (MAR), mineralized surface/bone surface (MS/BS) and bone formation rate/bone surface (BFR/BS) were quantified to assess bone formation and mineralization capacity (n = 6). GPX4-CKO: Dmp1^{Cre}; GPX4$^{fl/fl}$ mice. Data are represented as the mean ± SD. ns = no significant. ANOVA with post-hoc Tukey–Kramer test.

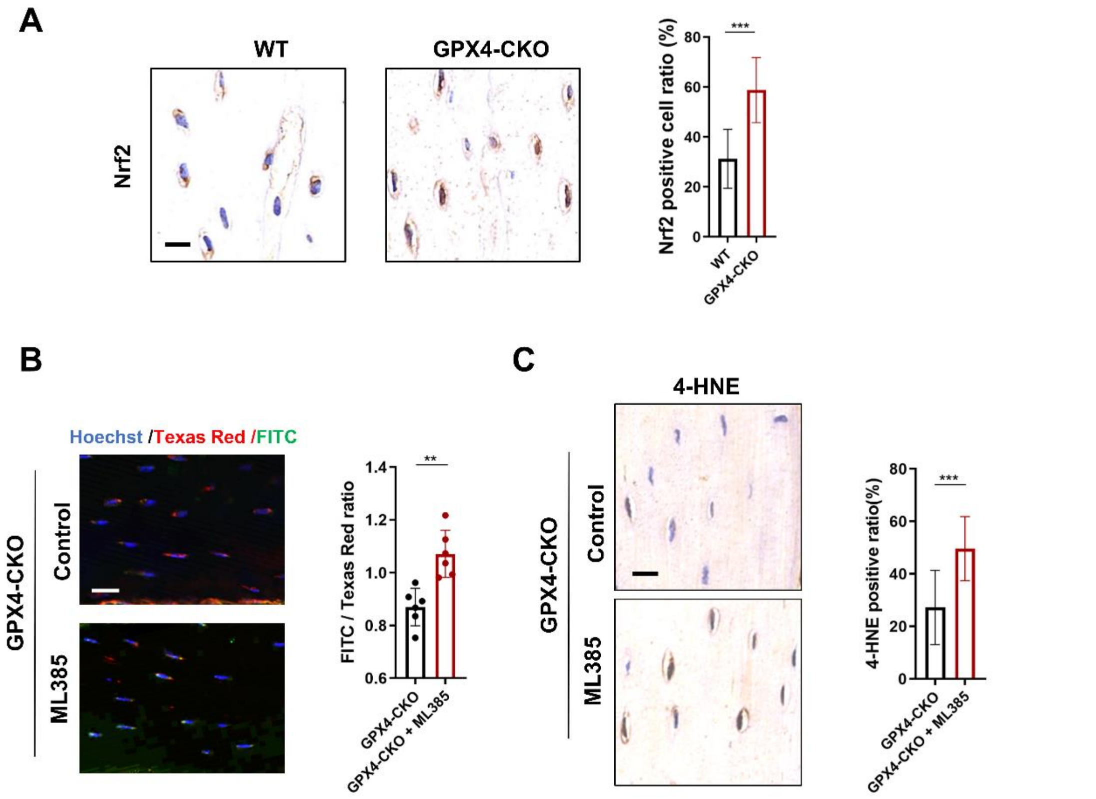

Figure S8. Nrf2 protect osteocytes from ferroptosis in GPX4-CKO mice. (A) Representative images of IHC staining for detecting Nrf2 in osteocytes of WT and GPX4-CKO mice and quantification (scale bar, 20 μm). Five randomly selected viewing fields were evaluated per section, and 6 mice were evaluated per group. (B) Lipid peroxidation was determined by C11-BODIPY 581/591 staining from GPX4-CKO mice treated with and without ML385. The fluorescence intensity of the green/red ratio was quantified (n = 6, scale bar, 20 μm, red: normal lipids; green: oxidized lipids; blue: cell nuclei). (C) Representative images of IHC staining for detecting 4-HNE in osteocytes of GPX4-CKO mice treated with or withou ML385 and quantification (scale bar, 20 μm). Five randomly selected viewing fields were evaluated per section, and 6 mice were evaluated per group. GPX4-CKO: Dmp1^{Cre}; Gpx4$^{fl/fl}$ mice. Data are represented as the mean ± SD. $^{**}P < 0.01$, $^{***}P < 0.001$. Two-tailed Student's t test.

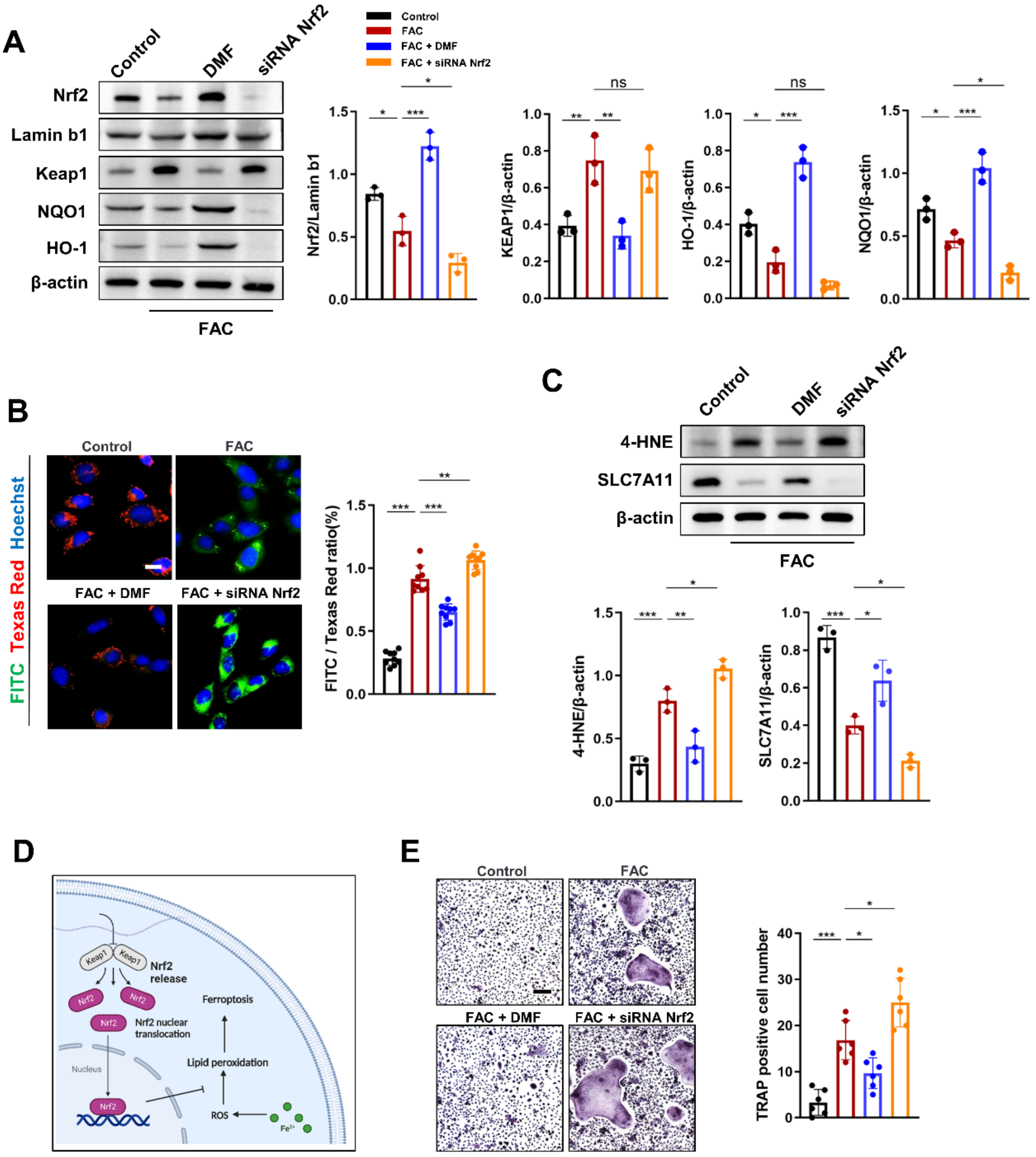

Figure S9. Nrf2 plays an important role in the regulation of ferroptosis in osteocytes and subsequent hyperactive osteoclastogenesis *in vitro*. (A) Representative Western blot and quantification of the protein level of Nrf2 in the nucleus and the protein levels of Keap1 and Nrf2 downstream genes, including NQO1 and HO-1, in Ocy454 cells (n = 3). (B) Ocy454 cells were stained with C11-BODIPY 581/591 and observed under a confocal fluorescence microscope. The fluorescence intensity of the green/red ratio was quantified to assess lipid peroxidation. (scale bar, 20 μm, red: normal lipids; green: oxidized lipids; blue: cell nuclei). Three randomly selected viewing fields were evaluated per sample, and 3 samples were evaluated per group. (C) Representative Western blot and quantification of the protein levels of 4-HNE, and SLC7A11 in Ocy454 cells (n = 3). (D) Schematic of Nrf2 function in the regulation of ferroptosis in osteocytes. (E) Representative pictures of TRAP staining (scale bar, 100 μm) of BMMs cocultured with Ocy454 cells and quantification of TRAP-positive

multinucleated (≥3 nuclei) cell number per well (n = 3). Data are represented as the mean ± SD. *P < 0.05, **P < 0.01, ***P < 0.001, ns = not significant. ANOVA with post-hoc Tukey–Kramer test.

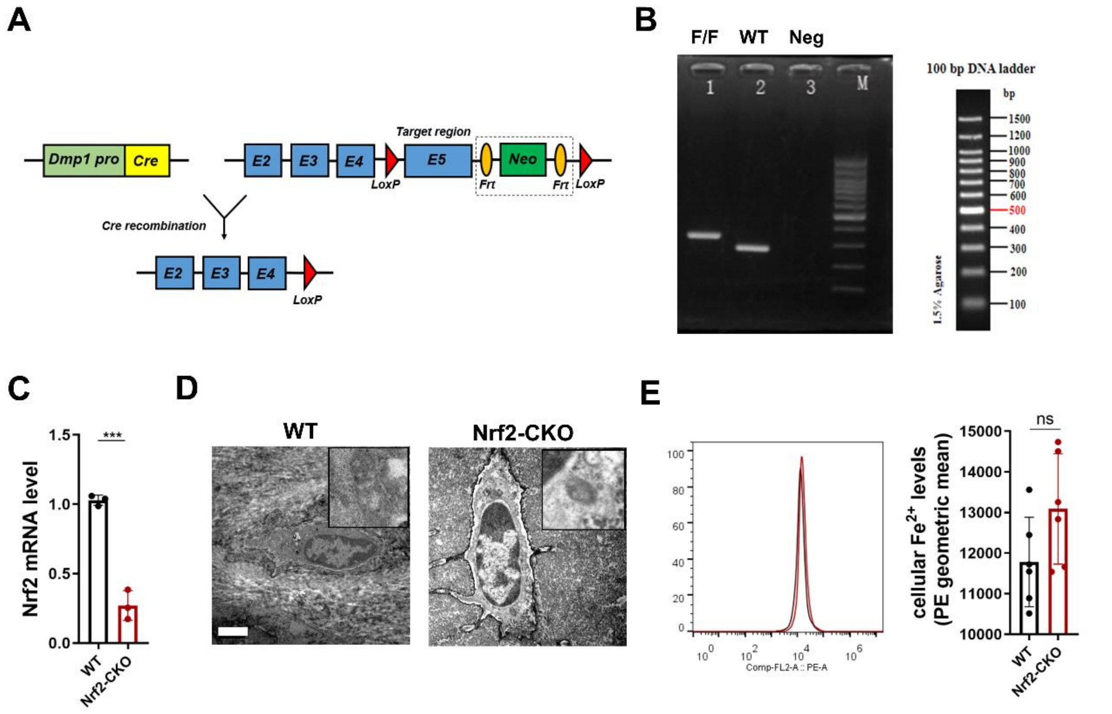

Figure S10. Dmp1^{Cre}; Nrf2$^{fl/fl}$ mouse construction and validation. (A) Dmp1^{Cre}; Nrf2$^{fl/fl}$ mouse construction strategy. (B) Genotyping of the Nrf2$^{fl/fl}$ and WT mice. (C) qPCR analysis of Nrf2 expression in the bone tissue (femoral shaft) of the WT and Nrf2-CKO mice (n = 6). (D) TEM was used to observe the morphological changes in mitochondria of osteocytes in the femur of WT and Nrf2-CKO mice (scale bar: 2 μm). (E) Fe^{2+} levels in osteocytes of WT and Nrf2-CKO mice were determined by FerroOrange staining and quantitation (n = 6). Nrf2-CKO: Dmp1^{Cre}; Nrf2$^{fl/fl}$ mice. Data are represented as the mean ± SD. ***P < 0.001, ns = not significant.Two-tailed Student’s t test.

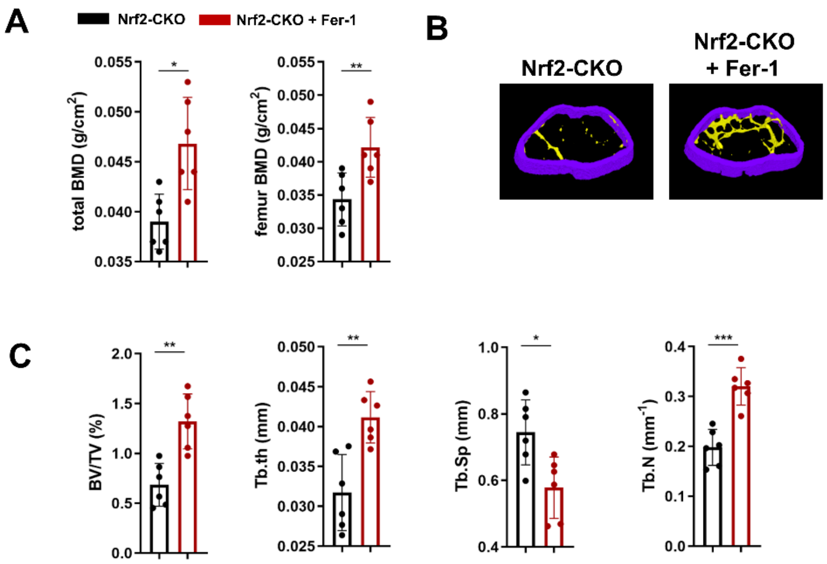

Figure S11. Ferroptosis inhibitor protected Dmp1^{Cre}; Nrf2$^{fl/f}$ mice from bone loss. (A) Total BMD and femur BMD of the Dmp1^{Cre}; Nrf2$^{fl/f}$ mice treated with and without Fer-1 (n = 6/group). (B) Representative μ-CT images of trabecular bone in the distal femurs (scale bar, 200 μm). (C) Distal femur BV/TV, Tb.th, Tb. N, and Tb.Sp were analyzed (n = 6/group). Data are represented as the mean ± SD. *P < 0.05, **P < 0.01, ***P < 0.001. Two-tailed Student's t test.

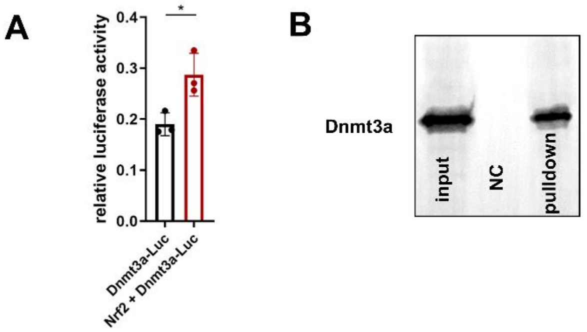

Figure S12. Nrf2 contributes to the transcriptional regulation of Dnmt3a. (A) Dual-luciferase reporter assays results showed Nrf2 promoted Dnmt3a transcription in osteocytes (n = 3). (B) DNA pull-down assay combined with western blotting analysis for the binding of Dnmt3a to RANKL gene promoter, respectively, in osteocytes using special DNA probes or negative control probe. Data are represented as the mean ± SD. *P < 0.05. Two-tailed Student's t test.
